# Amalur EIS: a system for calculating the environmental impacts of industrial sites from E-PRTR records

**DOI:** 10.1007/s10661-024-13565-3

**Published:** 2025-01-11

**Authors:** Iñaki Sasia, Gorka Bueno, Iker Etxano

**Affiliations:** 1 EKOPOL, Research Group On Ecological Economics and Political Ecology, Faculty of Social Sciences and Communication, University of the Basque Country (UPV/EHU), Sarriena S/N, 48940 Leioa, Basque Country Spain; 2 Department of Electronics Engineering, Faculty of Engineering of Bilbao, University of the Basque Country (UPV/EHU), Torres Quevedo Ingeniaria Plaza, 1, 48013 Bilbao, Basque Country Spain; 3 Department of Applied Economics, Faculty of Social Sciences and Communication, University of the Basque Country (UPV/EHU), Sarriena S/N, 48940 Leioa, Basque Country Spain; 4 HEGOA, Institute of Development Studies and International Cooperation, University of the Basque Country (UPV/EHU), Zubiria Etxea, Avda. Lehendakari Agirre 81, 48015 Bilbao, Basque Country Spain

**Keywords:** Amalur EIS, PRTR, LCIA, Environmental impact, Industrial activity

## Abstract

This article presents Amalur EIS (https://www.amalur-eis.eus/), an Environmental Information System that estimates environmental impacts using data sourced from the European Pollutant Release and Transfer Register database (E-PRTR). The system uses data on the releases into land, air and water of 31,556 European industrial facilities for the period 2007–2021. Amalur EIS calculates environmental impacts of industrial releases using 31 life cycle impact assessment methods (LCIA) and covering 78 of the 91 pollutants regulated by the PRTR Protocol. The system has been constructed using a two-layer software infrastructure: (i) a data layer supported by a relational database built in Postgres and (ii) a presentation layer built in Tableau, so it provides user-friendly access to the information. For an illustrative analysis of the tool, the EF 3.0 LCIA method recommended by the European Commission was used, including normalisation and weighting steps for a better comparison. The analysis concludes that the *climate change* impact category contributes the most (68.6%) to the total impacts, while the largest contributor from an economic activity perspective is the energy sector (59.5%). Geographically, both elements coincide in the German regions of Düsseldorf, Köln and Brandenburg, resulting in the concentration of the largest impacts at the European regional level. In fact, Germany is the country with the highest impact (20.3% of total). Beyond this analysis, Amalur EIS is poised to be a valuable tool for tracking the transition towards sustainability, particularly in Europe.

**Supplementary Information:**

The online version contains supplementary material available at 10.1007/s10661-024-13565-3.

## Introduction

Pollutant Release and Transfer Registers (PRTR) respond to the imperative of ensuring public access to information on environmental pollution. Their implementation is bolstered by various organisations and international agreements, aiming to collect and disseminate crucial information on the release and transfer of pollutants. The inaugural registry, the Toxics Release Inventory (TRI) (United States Environmental Protection Agency, 2023), emerged in the USA in 1986 following the catastrophic Bhopal disaster of 1984, one of the deadliest industrial incidents in history (Broughton, 2005; Varma & Varma, 2005). Key players such as the Organisation for Economic Co-operation and Development (OECD, 2023) and United Nations Conference on Environment and Development (UNCED) have played pivotal roles in the development and implementation of PRTRs. The OECD produced a guidance manual in 1996 (OECD, 1996), defining PRTRs as registries of releases and transfers of potentially harmful pollutants. The PRTR Protocol was adopted at an extraordinary meeting of the Parties to the Aarhus Convention in May 2003 and was signed by 36 countries and the European Community (UNECE, 1998). Supported by UNCED, this protocol has further promoted the implementation of PRTRs at the international level. Several countries have established national PRTR systems following the recommendations of UNCED and the OECD.

Alongside to the implementation of the PRTR Protocol, the European Union (EU) developed its own register, the European Pollutant Emission Register (EPER), regulated by Decision 2000/479/EC (European Commission, 2000), which evolved into the European Pollutant Release and Transfer Register (E-PRTR) in 2006 by the adoption of Regulation (EC) number 166/2006 (European Union, 2006). It was approved by the EU in February 2006, becoming the second party to sign the PRTR Protocol, after Luxembourg.

Covering 65 economic activities across Europe, the E-PRTR has been collecting comprehensive data on pollutant releases since 2007 and integrating it into the European Industrial Emissions Portal (European Environment Agency, 2023a). The E-PRTR establishes a list of 91 pollutants that certain industrial complexes and agricultural activities meeting specific size and activity criteria—around 35,000 installations—are required to report annually above certain emission or waste transfer quantities. The E-PRTR has improved its performance over the years to become a reliable and consistent database, despite difficulties such as systematising the reporting process, lack of information and occasional errors (Bünger, 2010; Dios et al., 2014; Kolominskas & Sullivan, 2004).

While PRTRs do not directly regulate emissions, they do exert pressure on companies to reduce pollution, creating incentives for emission reduction. Hamilton (1995) and Khanna et al. (1998) identified significant impacts when examining investor reactions to the public disclosure of TRI environmental information. Market penalties were also observed for firms with the highest levels of pollution in the EPER (Cañón-de-Francia et al., 2008), although only some emissions are directly regulated by the EU (e.g. CO_2_ emissions). Nonetheless, pollution datasets such as the E-PRTR do exert pressure on companies that want to avoid being identified as big polluters (Cañón-de-Francia et al., 2008; Hamilton, 1995; Khanna et al., 1998) and allow governments and interest groups to participate in decision-making and advocate for cleaner practises (Fikru, 2011; Zuo & Wheeler, 2019).

Public access to information on pollutant releases and transfers has become a pivotal tool for promoting transparency and accountability globally. PRTRs, epitomised by the E-PRTR, play a crucial role in pollution prevention and reduction, safeguarding the environment and human health on an international scale. The scientific literature includes studies on the use of PRTR registers in various geographic areas, such as the USA (Gouldson et al., 2015; Hamilton, 1995; Khanna et al., 1998; Koh et al., 2016; Thant Zin & Lim, 2023), Japan (Nguyen et al., 2021), Australia (Zuo & Wheeler, 2019), Europe (Assen et al., 2016; Bünger, 2010; Cañón-de-Francia et al., 2008; Fikru, 2011; Kolominskas & Sullivan, 2004; Pistocchi et al., 2019; Shaddick et al., 2018) and within it, Austria (Rüttenauer, 2018), Sweden (Nordborg et al., 2017; Sörme et al., 2016), Spain (Fernández-Navarro et al., 2017; García-Pérez et al., 2013) and its region of Galicia (Dios et al., 2012, 2014).

PRTR databases have been used to evaluate different ways in which pollution impacts the environment (Dios et al., 2012). Several studies evaluate its impact on human health: Shaddick et al. (2018) evaluate the health impact of pollution from landfills; Fernández-Navarro et al. (2017) analyse the role of air pollution on cancer; García-Pérez et al. (2013) analyse cancer mortality in populations close to incinerators and hazardous waste recovery or disposal facilities. Some studies focus their analyses on raw emissions data (Dios et al., 2012), or other environmental impacts. Assen et al. (2016) use E-PRTR records to determine the environmental impacts of CO_2_ capture, while Pistocchi et al. (2019) quantify river pollution. Other studies focus their work on toxicological footprints. Sörme et al. (2016) calculate the national chemical footprint of Sweden. Koh et al. (2016) analyse the toxicological footprint for the chemicals in the TRI release. Nguyen et al. (2021) conduct an analysis of toxicological footprint changes in Japanese industrial sectors, and Erhart and Erhart (2022) provide a Swedish human toxicity and ecotoxicity footprint analysis, complemented with an environmental ranking of European industrial facilities by toxicity and global warming potentials (Erhart & Erhart, 2023). E-PRTR records have also been used to investigate the extent of environmental inequality related to industrial air pollution in Austria (Neier, 2021).

Through the European Industrial Emissions portal (European Environment Agency, 2023a), the flows of pollutants emitted by industries reporting to E-PRTR can be consulted from various analytical perspectives. However, the E-PRTR does not offer a direct translation of these simple metrics in kg units to complex indicators in units such as kgCO_2_eq, CTUe or molH^+^eq, linked to the different environmental impact (EI) categories established by the numerous existing life cycle impact assessment (LCIA) methods. Several studies have made progress in quantifying impacts. Nguyen et al. (2021) and Koh et al. (2016) employ a *toxicological footprint* indicator, which is just the sum of masses of the different pollutants, without applying a toxicity factor. This drawback has been solved by some others (Nordborg et al., 2017; Sörme et al., 2016) that apply characterisation factors from the USEtox LCIA method in their calculations of Sweden’s chemical footprints. Thant Zin and Lim (2023) propose MECPRIA, a methodology to evaluate the toxicity of PRI pollutant releases, which is an alternative to quantity-based (Koh et al., 2016) and LCIA-based (Sörme et al., 2016) methodologies. When using LCIA methods, they also rely on the USEtox LCIA method. Very recently, the EEA has estimated the external costs of industrial air pollution trends (2012–2021) from E-PRTR facilities, calculating the marginal damage cost on human health due to pollutants (European Environmental Agency, 2024). A report by Mawdsley et al. (2016) commissioned by the Swedish Environmental Protection Agency also identified the use of weighted aggregations to properly account for pollutant impacts, such as toxicities, as a central issue.

Amalur EIS has been developed with the aim of advancing in the translation of pollutant emissions into the quantification of impacts (*Amalur* means *Mother Earth* in Basque language). It is an Environmental Information System (EIS) with which to calculate the EI of the emissions gathered in the E-PRTR for the different impact categories proposed by several LCIA methods, such as follows:

- BEES + (NIST, 2023)
- CML-IA [Baseline] [Non-baseline] (CML, 2023)
- Crustal Scarcity Indicator (Arvidsson et al., 2020)
- Cumulative [Energy Demand] [Energy Demand (LHV)] [Exergy Demand] (Hischier et al., 2010)
- Ecological Scarcity 2013 (Frischknecht & Knöpfel, 2014)
- EDIP 2003 (Danish Ministry of the Environment, 2005)
- EF Method (Adapted) [2.0] [3.0] (Joint Research Centre, 2023)
- EN 15804 + A2 Method (EPLCA, 2023a)
- Environmental Prices (CE Delft, 2023)
- EPD 2018 (EPD International, 2023)
- EPS 2015 [D] [DX] (IVL, 2023)
- ILCD 2011 Midpoint + (EPLCA, 2023b; JRC, 2012)
- Impact 2002 + (Jolliet et al., 2003)
- IPCC [2013] [2021 AR6] (Forster et al., 2021)
- ReCiPe 2016 [Midpoint (E)(H)(I)] [Endpoint (E)(H)(I)] (Huijbregts, 2016)
- Selected LCI [Results] [Results, Additional] (Hischier et al., 2010)
- TRACI 2.1 (J. Bare, 2011; J. C. Bare et al., 2002)
- USEtox 2 [Recommended Only] [Recommended + Interim] (Rosenbaum et al., 2011)

These LCIA methods provide diverse geographic, sectoral and impact-specific perspectives, making assessments more comprehensive and regionally relevant. Methods like BEES + and TRACI 2.1 are tailored to North American priorities, while EF aligns with European standards and regulation. Some methods offer a broad scope of impacts, like CML-IA, ILCD, EF and ReCiPe, which cover multiple categories. Others, such as IPCC for *climate change* and USEtox for *toxicity*, provide specialised analyses in their domains. Resource-focused methods (Crustal Scarcity Indicator, Ecological Scarcity 2013) and energy-demand metrics (*cumulative energy* and *exergy demand*) address specific sustainability issues, while economic-based approaches (Environmental Prices, EPS 2015) provide cost-focused insights. Endpoint approaches (EPS 2015, ReCiPe) offer aggregated results ideal for decision-making, while midpoint methods (CML-IA, ILCD, EF) provide detailed impact profiles. Using multiple methods enables cross-validation, which strengthens the results and reveals areas needing further analysis.

In each case, the EI contributions to air, water and land pollution are calculated by Amalur EIS, and for some pollutants, their release is also computed as resource depletion. There are other tools that also process PRTR data. For example, Overberg et al. (2023) developed a Python-based tool for data analysis and visualisation, and Dios et al. (2014) created a software tool for the validation of E-PRTR emissions data. Unlike these applications, Sörme et al. (2016) go a step further in their chemical footprint calculation by converting the emissions collected in the E-PRTR into categorised EIs. To our knowledge, it is the first published work that does so, although it only uses USEtox from among the various LCIA methods. To fill this gap, Amalur EIS provides a comprehensive set of LCIA methods and EIs, as indicated above, making it a robust and multidimensional tool. The conversion of emissions into environmental impacts represents a step forward in environmental information systems, as it facilitates the identification of impacts beyond pollutants through various indicators.

The primary objective of this work is to demonstrate the potential of Amalur EIS in estimating the environmental impacts of emissions from industrial facilities, providing ecological indicators that are not directly available from the raw data of PRTR records. The article is structured as follows: the “Methods and materials” section details the main design criteria used in developing the software; the “Results” section presents significant findings related to emission volumes, analysis by impact categories, sectors of activity and geographical scope; and the “Discussion and conclusions” section outlines and discusses the key contributions of Amalur EIS in the field of ecological indicators.

## Materials and methods

Amalur EIS relies on a two-layer software infrastructure: a data layer supported by a relational database built in a Postgres (PostgreSQL, 2023) RDBMS (Relational DataBase Management System) and a presentation layer built in Tableau (Tableau, 2023) providing a UI (User Interface) to facilitate exploitation of the information.

### Data layer construction process

The process of building the data layer required the prior design of an ERM (entity relationship model) to house all the data necessary to meet the desired informational objectives. Subsequently, this ERM was physically implemented in a Postgres RDBMS to finally perform the corresponding data ingestion from the starting sources, essentially E-PRTR and LCIA methods in openLCA format.

#### Emissions in E-PRTR

E-PRTR v18 is a relational database implemented in a Microsoft Access v2016 RDBMS that can be downloaded from the (European Environment Agency, 2023b). Using SQL (Structured Query Language) code, queries (views, logical tables) are generated that fit directly into the physical table structure built in Postgres. Thus, the information incorporated into Amalur EIS in this phase is simply a list of all the emissions reported by the industrial complexes over the years 2007–2021 (hereafter, years always refer to years of pollutant emissions and not to their being reported several years later). The database also processes diffuse air emissions for the year 2008, currently the only year available for said emissions in the E-PRTR. A more detailed analysis of these diffuse emissions is reserved for a future publication. At present, the database does not include information on off-site transfers of waste, but its structure is designed to accommodate such data in future extensions of the tool.

The only significant problem encountered in this implementation phase is that practically one third of the industrial complexes located on land, as well as all those located offshore, have their NUTS field empty. Since this will be a critical field when carrying out impact analysis from a geographical perspective, we succeeded in solving this problem by intersecting the E-PRTR layer of points (lat, long) of industrial complexes with the polygon layer of NUTS regions (Eurostat, 2023). The result of the corresponding spatial overlap query is as follows:

- Onshore facilities (93.6%) inherit the NUTS code of the region that contains them (their distance to it will always be 0 km).
- Facilities in territorial waters (5.7%) inherit the NUTS code of the nearest land region (their distance to it will always be less than the 22.2 km equivalent to the 12 nautical miles established for the limit of international waters).
- Facilities in international waters (0.6%) are assigned a NUTS code linked to the generic Extra-Regio region of their corresponding country with code *zzz* (their distance to it will always be greater than 22.2 km).

#### Characterisation factors of LCIA methods

The characterisation factors (CF) of the LCIA methods considered were obtained from the *openLCA LCIA Methods (v2.2.1) database*, accessible through Nexus (2023). This resource is a relational database implemented in an Apache Derby v10 RDBMS, used by the openLCA software (Ciroth, 2007). Similar to the process described above, a selection of its data is imported into the physical table structure built for Amalur EIS. In this case, the information incorporated into the system is related to issues such as LCIA methods and their impact categories, elementary flows and what is needed to quantify their impact in terms of the environment, equivalent units of measurement, etc.

In the process of preparing the data in Apache Derby for subsequent ingestion into the final Postgres repository, no significant problems were found. The most significant issue that arose is that some elementary flows have slightly different names in different LCIA methods, sometimes such minor details as the use of a hyphen, a space or a comma and the use of upper- or lower-case letters. These instances have required specific handling when loading characterisation factors into Amalur EIS.

The Table SM1 “Characterisation factors” provided in the Supplementary Material presents the complete list of pollutants included in the E-PRTR protocol, with their corresponding characterisation factor for each medium and each impact category of the Environmental Footprint (EF) 3.0 LCIA method.

#### The union of emissions and environmental impacts in Amalur EIS

Amalur EIS v2024 is a relational database implemented in a Postgres v15 RDBMS. As explained, it hosts a set of tables linked to the E-PRTR domain and another set of tables linked to the LCIA methods domain. In addition, it includes a third set of ad hoc built-in tables that close the unified ERM by linking the two previous domains. These linking tables constitute a major contribution of this research and form the real heart of the system: they link every pollutant in the E-PRTR releases database with a specific elementary flow in each LCIA method, if its characterisation factor is available.

The LCIA methods characterise the elementary flows given in the life cycle assessment (LCA) methodology (ISO, 2006a, b) by means of characterisation factors based on three fundamental elements: pollutant, medium and environment.^1^ Thus, to link the information contained in the E-PRTR domain with that provided by the LCIA methods domain, it is necessary to establish one-to-one relationships at these three levels between the table records of the two informational environments. The definition of such relationships at the database level was met with several problems that were finally resolved using the methodology detailed in the following sections.

##### Linking < E-PRTR pollutant > with < elementary flow from LCIA methods > 

The most obvious candidate, a priori, for a key to link the pollutants typed in E-PRTR with those included in the LCIA methods flows was the CAS number (Chemical Abstracts Service) (American Chemical Society, 2023). However, after an initial analysis, it was found that, although this field exists in the LCIA methods flow table, 10% of the records are missing this information. On the other hand, a considerable number of pollutants collected in E-PRTR are not assimilable to a specific CAS, such as halogenated organic compounds, chlorides or particulate matter, to name but a few. Thus, this double circumstance de facto rules out the potential use of the CAS as a binding key, making it necessary to define an artificial key for this purpose.

This key was established semi-automatically by implementing successive search processes that, cumulatively, have made it possible to link more and more records from both domains. Thus, in an initial approximation, all direct CAS matches between < E-PRTR pollutant > and < elementary flow from LCIA methods > were identified. As already known, this has only worked satisfactorily in simple cases such as that of methane, which is clearly typed in the E-PRTR as *CH*_*4*_ and correctly labelled as *74–82-8* in most of the LCIA method flows. Next, we searched for literal matches by name, again finding several matches in simple cases such as that of carbon dioxide. From there, it was necessary to conduct a multitude of further searches, this time guided by new criteria, no longer of literal matches but of mere similarity, and not only at name level but also at that of other possible labels such as chemical formula and acronym. Finally, a priority order was established so as to be able to choose a single characterisation factor (CF) in the case that several CF can be linked to the same emission registered in E-PRTR. The criterion used to establish this hierarchy was specific for each pollutant. In most cases, the hierarchy is irrelevant, since differences are simply due to name variations (*Nonylphenol*, *NONYLPHENOL*; *Hydrocyanic acid*, *Hydrogen cyanide*). In the case of *Carbon monoxide*, for example, the *Carbon monoxide* elementary flow was always chosen first, or otherwise the *Carbon monoxide, fossil*, or otherwise the *Carbon monoxide, land transformation*, or otherwise the *Carbon monoxide, biogenic*. In the case of metals, the approach proposed by Sörme et al. (2016) was adopted, taking the compound species with the highest CF (most toxic). Thus, for *Chromium and compounds (as Cr)*, for example, the chosen hierarchy was *Chromium*, *Chromium, ion*, *Chromium compounds* and *Chromium, unspecified*. The same procedure was followed for other metals.

**Fig. 1:**
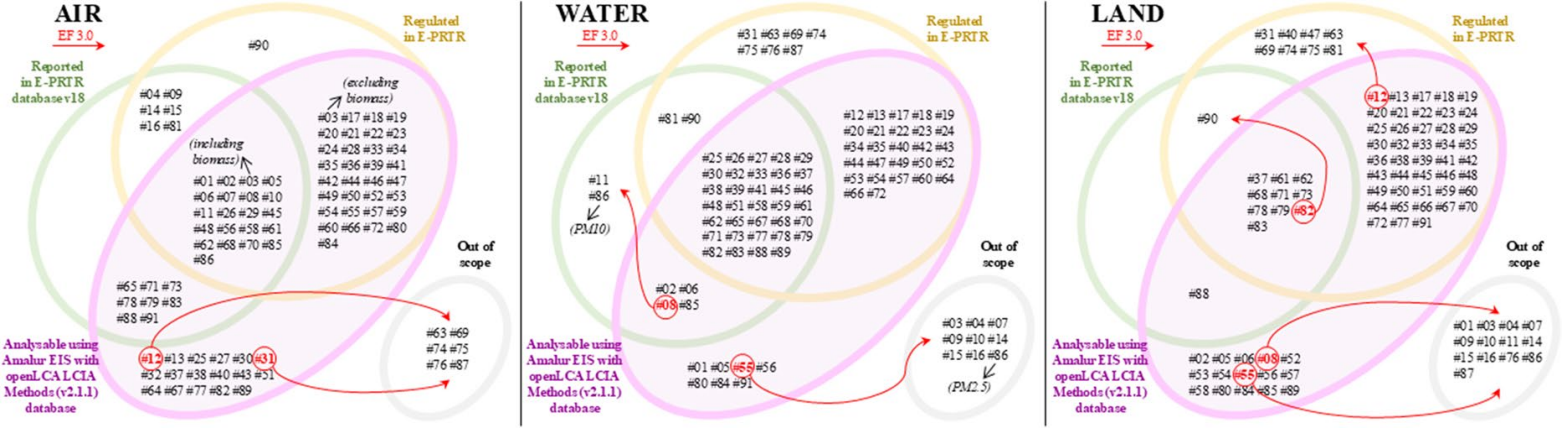
Scope of Amalur EIS v2024. Source: Amalur EIS

In any case, the result of this rigorous process was highly satisfactory, since 86% of the pollutants typified in E-PRTR have finally been linked to their corresponding elementary flow covered by at least one LCIA method (and usually by several). Thus, Amalur EIS is currently able to quantify the EI of the emissions linked to 78 of the 91 pollutants recorded and is only limited for the following 13 pollutants that are left out: hydro-fluorocarbons (HFCs) [#04], perfluorocarbons (PFCs) [#09], hydrochlorofluorocarbons (HCFCs) [#14], chlorofluorocarbons (CFCs) [#15], halons [#16], brominated diphenylethers (PBDE) [#63], organotin compounds (as total Sn) [#69], tributyltin and compounds [#74], triphenyltin and compounds [#75], total organic carbon (TOC) (as total C or COD/3) [#76], asbestos [#81], octylphenols and octylphenol ethoxylates [#87] and hexabromobiphenyl [#90]. Furthermore, these results are even more promising considering that only 7 of these 13 pollutants (namely, [#04], [#09], [#14], [#15], [#16], [#81] and [#90]) are reported by any facility in E-PRTR v18, which puts Amalur EIS at 92% effectiveness (see Fig. 1, which shows the scope of Amalur EIS v2024 for all 24 LCIA methods considered; red arrows show the scope variation when only the EF3.0 LCIA method is considered).

Figure 1 shows the scope of the tool in terms of its capacity to identify release flows. The area labelled *Analysable using Amalur EIS with openLCA LCIA Methods (v2.2.1) database* contains all the flows that Amalur EIS can analyse in at least one of the 24 LCIA methods considered. On the other hand, red arrows indicate which of these releases are not analysable in the specific case of the EF 3.0 LCIA method. As seen, Amalur EIS fails to analyse the impact of eight flows involving five pollutants ([#08], [#12], [#31], [#55] and [#82]). Most of these cases do not really constitute a limitation, as they are neither regulated nor reported. Pollutant [#08] into water is equally irrelevant, since it is not regulated, although it is reported, while pollutant [#12] into land could become a limitation, although it is true that no facility has reported it so far. Thus, pollutant [#82] into land is the only case that constitutes a real limitation, since it is a regulated and reported pollutant whose impact Amalur EIS is unable to calculate using the EF 3.0 LCIA method.

The final scope of the system in its current version (v2024) at the level of pollutants, media and LCIA methods is provided comprehensively in Table SM2 “Exhaustive scope of Amalur EIS v2024”, under Supplementary Material.

##### Linking < medium E-PRTR > with < medium LCIA methods > 

In this case, there was no difficulty in identifying the medium, since the name matching between < medium E-PRTR > and < medium in LCIA methods > is straightforward, except in the case of the *soil* and *land* tags, which are very easily taken as equivalent, in any case.

##### Linking < environment E-PRTR > with < environment LCIA methods > 

The fact that LCIA methods assign CF to elementary flows according to medium and environment means that the quantification of the EI caused by the emission of a given quantity of pollutant into a particular medium varies depending on the environment in which it occurs. This being so, to complete the process of total and definitive unification of the E-PRTR and LCIA method domains, it was absolutely essential to correctly link this third and final element. However, it quickly became apparent that the challenge would be impossible to overcome by any simple means: no E-PRTR table contains any field that can be even remotely taken as equivalent to the concept of *environment*. Faced with such a scenario, the only plausible option was to establish a criterion that allows one and only one of all the possible flows associated with a particular pollutant and medium in its various environments to be associated with the emissions recorded in E-PRTR. For this purpose, an order of priority was defined as shown in the following hierarchy:

- Air: Unspecified > high population density > low population density > lower stratosphere + upper troposphere > indoor
- Land: Unspecified > industrial > agricultural > forestry
- Water: Unspecified > freshwater > river > lake > ocean > surface water

The *unspecified* environment was chosen whenever available. If not available, for the *air* and *land* media, it was assumed that industrial facilities tend to be located in *industrial* areas with a *high population density*. For the *water* medium, the *freshwater* environment was prioritised, following Sörme et al. (2016). A sensitivity analysis was carried out to evaluate the significance of using *unspecified* as the first option versus its use as the last option in the hierarchy. When calculating total impacts for all E-PRTR records using the EF 3.0 LCIA method after normalisation and weighting, this change resulted in an increase in total impacts of only 0.8%, allowing us to conclude that its effect is limited.

Finally, it should be noted that the LCIA methods database takes a fourth medium *resource* into account, in addition to the three of *air*, *land* and *water*. This fourth medium is related to the elementary flows of the impact categories associated with resource depletion. Therefore, the E-PRTR releases were also linked to these elementary flows for their computation in the respective resource depletion impact categories.

### Presentation layer construction process

When exploiting a database, it is advisable to build a presentation layer, that is, a user-friendly UI that abstracts from the data layer and focuses on the informational potential of the created system. In this regard, E-PRTR makes use of a shallow interaction interface through the European Industrial Emissions Portal (European Environment Agency, 2023a), but the data exploitation capabilities of this platform are highly limited.

**Fig. 2:**
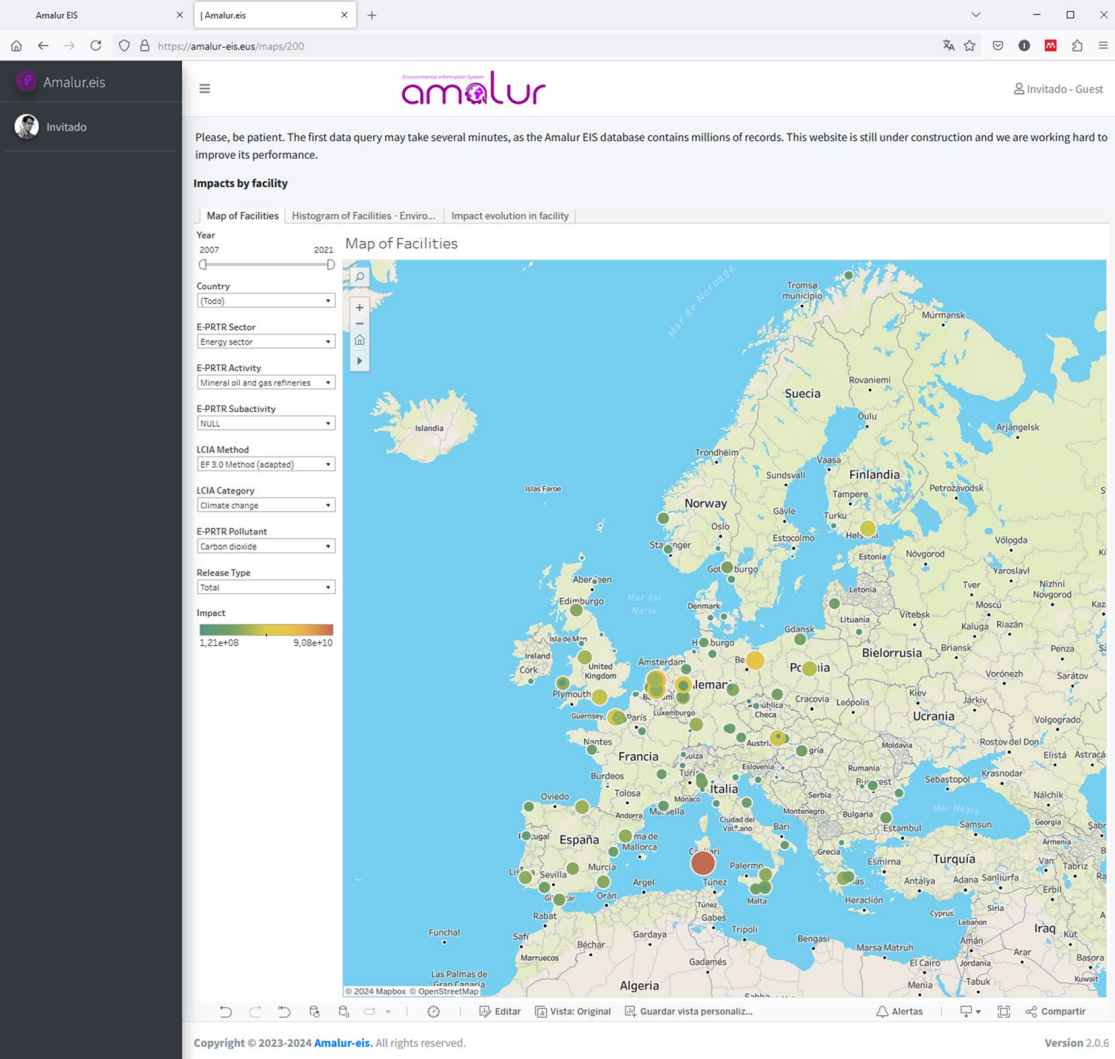
Amalur EIS web site. Source: https://www.amalur-eis.eus/

In the case of Amalur EIS, a data exploration environment has been created with Tableau v2022 that allows the end users of the system to benefit from all the power of an information system via a simple, responsive and visually intuitive interface (see Fig. 2).

## Results

Amalur EIS performs complex multidimensional analyses, yet the results are presented in a very visual and easily interpretable way, through coloured geographical maps or simple histograms. The information system itself and its usefulness as a tool is the main result of the work carried out. Moreover, from its usage, other results are derived, demonstrating the practical value of the tool. This is what is shown in the following sections, presenting a large number of use cases with remarkable results that show the potential of the software in response to particular queries. In most cases, the EF 3.0 LCIA method has been used. In fact, EF is the LCIA method proposed by the European Commission (2021) to measure and communicate the life cycle environmental performance of products and organisations. EF is an extensive method which covers 77 pollutants out of the 91 regulated by E-PRTR in a wide variety of impact categories (26 in total, related to *acidification*, *climate change*, *ecotoxicities* and *human toxicities*, *eutrophication*, *ozone depletion*, *particulate matter*, *photochemical ozone formation* and *resource use*). Thus, this section is divided according to the particular analyses conducted regarding release volumes, impact categories, activity sectors and geographical scope.

### Findings related to release volumes

Before analysing LCA indicators, two facts must be highlighted, observed when using Amalur EIS directly on the basic metrics provided by E-PRTR. The first issue is shown in Table SM3 “Top 100 release outliers in E-PRTR” provided in the Supplementary Material, which ranks the 100 facilities with the highest volume of releases. In order to determine outliers within a sample, it is common to consider values below q1 − 1.5 × (q3 − q1) or above q3 + 1.5 × (q3 − q1) as outliers (where q = quartile). Analysing the records reported in E-PRTR database in this way, Amalur EIS detects 5.46% abnormal releases (30,028 outliers in 549,545 records of pollutant releases), of which the 100 given in Table SM3 are striking, since they are over 100 times greater than the median of pollutant releases reported in the period 2007–2021 by a single facility, taking only controlled reports into account (accidental reports have not been included in the analysis so as not to distort the results). It can be confirmed that the top 11 releases listed in Table SM3 exceed the median by thousands of times and can only be interpreted as reporting data quality errors, particularly the first two, whose order of magnitude is several million times the median. It must be taken into account that a non-negligible part of the data collected in E-PRTR may require corrections, as already pointed out by Dios et al. (2014) and Erhart and Erhart (2023). Moreover, Annex 4 of a Technical note recently published by the European Environmental Agency (2024) includes 228 corrections made to E-PRTR data concerning erroneous air release entries registered by 188 facilities. Of these corrections, 40% (specifically 88 of 228) had already been identified as abnormal releases by Amalur EIS using the aforementioned outlier assessment method based on quartile analysis, which identifies values that deviate markedly within a given sample. In fact, two erroneous releases reported by the EEA also appear in Table SM3: DJP—De HoopBV, with 1.26 × 10^8^ kg of nitrogen oxides erroneously reported in 2021 (869 times the median), and ENIPOWER S.P.A.—Stabilimento di Brindisi, with 1.27 × 10^7^ kg of nitrous oxide erroneously reported in 2020 (326 times the median). The other 86 cases identified by Amalur EIS also meet the condition of outliers, although they do not appear in Table SM3 because they exceed the median by less than 100 times. Amalur EIS detects other outliers that are not included in Annex 4. The facility EDAR EL PRAT DE LLOBREGAT ranks 7th in Table SM3 for a Simazine release in 2014 that is multiplied by ten but is missing in Annex 4, probably because it is a release into water. The facility ranking 11th, EXPLOTACIÓN PORCINA (UNIFICADO) HERMANOS MATAS DE HUERTA S.L., presents an outlier methane release into air in E-PRTR that is clearly erroneous, as it does not appear in the Spanish PRTR. Another CO_2_ release from BIZKAIA ENERGIA, S.L. (a natural gas combined cycle) is not listed in Annex 4, although it is clearly erroneously reported as tenfold (PRTR España, 2024).

The second issue to highlight is that of trends over time. Notably, annual releases generally show a decreasing pattern, as indicated by the slopes of trend lines calculated from linear regression for each pollutant over the period 2007–2021 (refer to Table SM4 “2007–2021 E-PRTR Release Trend by Pollutant” in the Supplementary Material). However, we must be cautious before drawing hasty conclusions when interpreting the results of a large-scale data analysis. In this sense, it must not be forgotten that, while linear regressions with a *p*-value close to 0 will be highly accurate and predict with certainty the sustained evolution over time, those with a *p*-value close to 1 will be less accurate. Here, Amalur EIS is again a helpful tool, as it allows us to visualise the trend analyses performed (see Figure SM1 “Graphical extract of 2007–2021 E-PRTR releases trend by pollutant” provided in the Supplementary Material). This can be seen very clearly in the cases of carbon dioxide, nitrogen oxide or sulphur oxide releases into the air, whose *p*-value is < 1 × 10^−4^. In the case of toluene releases into water, the *p*-value increases to 1.87 × 10^−3^, but the trend line still demonstrates a relatively high predictive capability. On the other hand, the predictive relevance of the trend lines with *p*-values > 0.9 is very poor, as in the case of atrazine or chlordecone releases into water, or toluene releases into the air.

Amalur EIS allows for analyses such as those described above at the facility, activity sector and geographic region level. An example of this process is provided in Figure SM10 “Düsseldorf total releases during the period 2007–2021” in the Supplementary Material. The chosen case refers to releases per pollutant reported in Düsseldorf (Germany) by facilities in the energy sector, grouped by sub activity and NUTS 3 region. The emissions are represented on their own scale for each pollutant, so the size of the coloured rectangles is comparable between different pollutants only vertically, but not horizontally. In addition, the figure illustrates how easy it is to access the data underlying the graphical views by right-clicking the mouse to activate the contextual menu. In turn, this data can be easily exported for post-processing through other third-party tools external to Amalur EIS, such as SQL, Excel, SPSS or QtiPlot.

### Findings related to impact categories

The E-PRTR database allows us to consult isolated pollutant emission volumes but does not consider their combined impact. In contrast, Amalur EIS allows the user to total the impacts linked to the variety of pollutants that may be involved in each of the different impact categories. This is shown, for instance, in Table SM1 “Characterisation factors”, containing all the CFs of the E-PRTR regulated pollutants in each of the impact categories in the EF 3.0 LCIA method, which considers up to 69 different regulated pollutants in the *freshwater ecotoxicity* impact category. Amalur EIS makes it possible to calculate the total environmental impact (EI) in any specific category associated with the emission of all pollutants registered within a facility, region or activity sector in the E-PRTR, for a selected range of years. As an example, Table 1 shows the total EI associated with each of the activity sectors included in the E-PRTR for the entire period 2007–2021, in each of the impact categories included in the EF 3.0 LCIA method.

**Table 1:**
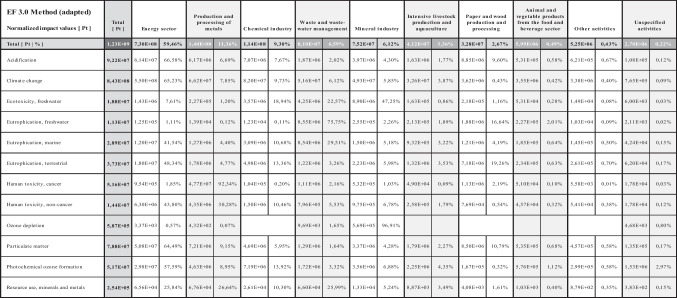
Total impacts by activity sector in each impact category of the EF 3.0 LCIA method, for the 2007–2021 period. Total impact for each activity sector (Pt units and % in the first row) is calculated after applying normalisation and weighting steps for the EF 3.0 LCIA method

Source: Amalur EIS

The processing of the 549,545 releases from the 31,556 facilities of the E-PRTR during the period 2007–2021 allows us to estimate, for example, a total impact of 32.4 Gt CO_2_ in *climate change*, 19.1 Mt Neq in *eutrophication (marine)*, 220,000 CTUh in *human toxicity* or 499 t CFC11eq in *ozone depletion*. The energy sector leads the impacts not only in the *climate change* category but also in *acidification*, *eutrophication*, *human toxicity (non-cancer)*, *particulate matter* and *photochemical ozone formation*. The intensive livestock production and aquaculture sector generates significant impacts in *eutrophication (freshwater)* (17% of the total) and *eutrophication (terrestrial)* (19%). The mineral industry accounts for 97% of the impact in *ozone depletion* and 47% in *ecotoxicity (freshwater)*.

Another of Amalur EIS’ potentialities is that it brings out divergences between different LCIA methods. For example, significant differences are observed between the *freshwater ecotoxicity* impact categories of EF 3.0 and USEtox 2 methods. Figures SM2 and SM3 in the Supplementary Material show the ranking for Spanish facilities (2007–2021) in the *ecotoxicity (freshwater)* category for the EF 3.0 and the USEtox 2 (recommended + interim) methods, respectively. The rankings differ significantly. The analysis of this specific case with our EIS shows that while impacts in USEtox 2 are dominated by copper and zinc compounds (87.1% and 7.2% of total), no impact is assigned to chlorides, which account for 88.9% of total impacts in EF 3.0. These results are consistent with Sala et al. (2022), who state that USEtox 2 presents a dominance of metals and lacks robust fate modelling for non-organic compounds.

### Findings related to activity sectors

Using Amalur EIS, information has been obtained on the impact of the different activity sectors included in the E-PRTR in the various impact categories proposed by the EF 3.0 LCIA method. Two visualisation examples are provided in the Supplementary Material. Figure SM4 “Impacts of the E-PRTR during the period 2007–2021 by activity sectors in the eutrophication terrestrial category” shows a histogram dominated by the energy sector (with an impact of 8.58 × 10^10^ mol N eq out of a total impact of 1.78 × 10^11^ mol N eq for the period 2007–2021) and the intensive livestock production and aquaculture sector (with an impact of 3.42 × 10^10^ mol N eq). And Figure SM5 “Map of regional (NUTS 2) impacts for the category eutrophication, terrestrial in the intensive livestock production sector” shows that only the three regions with the highest impact, Lombardia in Italy, Cataluña and Castilla y León in Spain, account for 4% of the total impacts (all sectors) in the E-PRTR in this category.

The role of each sector in each impact category in the EF 3.0 method is gathered in Table 2, which shows the percentage of the total impact (line 1) corresponding to each activity sector, as well as the percentage contribution to the sector impact (line 3) of the main pollutant involved (line 2). The quantity of pollutants appears in the second header line (72 different pollutants for *freshwater ecotoxicity total*, 60 for *human toxicity [non-cancer] total* and so on).

**Table 2:**
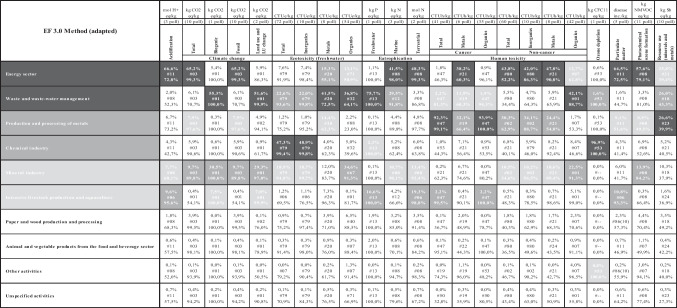
Impacts (%) by category (EF 3.0) in each activity sector (2007–2021)

Source: Amalur EIS

The highlighted entries reflect the three cases with the highest percentage impact by category; dark gray reflects the case with the highest percentage impact, gray the next highest and light gray the third highest

Amalur EIS reveals that the dominant pollutants vary according to the activity sector. Although it is true that there are certain categories, such as *climate change (biogenic)*, *freshwater ecotoxicity (metals)*, *eutrophication (freshwater)* and *ozone depletion*, in which the main pollutant is always the same ([#01], [#20], [#13] and [#53], respectively), this is not the case in many other categories, the two most significant cases being *freshwater ecotoxicity (organics)* ([#01], [#07], [#32], [#40], [#67], [#71] and [#88]) and *human toxicity (cancer)* ([#19], [#21], [#47], [#50] and [#53]) which show notable variations depending on the activity sector.

Activity sectors show significantly homogeneous behaviour at the level of impact in the distinct categories. The energy and waste sectors prove to be the most polluting in practically all the EF 3.0 categories, while those related to food and animal products and paper and wood production are the least polluting ones.

Amalur EIS also allows endpoint impact analysis. As an example, Table 3 shows the impacts provided by the ReCiPe 2016 Endpoint (I) method in the area of protection of ecosystem quality. When comparing these results with those provided by EF 3.0 (normalisation and weighting, first row of Table 1), we observe that the energy sector remains as the one with the highest impact (63.8% of total impact in ReCiPe; 59.5% in EF). But changes occur in the following positions: the energy sector is now followed by the mining sector (with 9.7% of total impact; 6.1% in EF) and the production and processing of metals (7.0%; in second position in EF with 11.4% of total impact). The ranking also changes in the area of protection of human health (energy sector, first place; mining, second and waste management, third). This can be checked in Figure SM6 “Ecosystem impacts by category (ReCiPe 2016 Endpoint (I)) in each activity sector (2007–2021)” in the Supplementary Material which extends the scope of Table 3 to the three areas of protection (human health, ecosystem quality and resource scarcity).

**Table 3:** Ecosystem impacts (species·year) by category (ReCiPe 2016 Endpoint (I)) in each activity sector (2007–2021)

ReCiPe 2016 Endpoint (I) (species·yr)	Total	Global warming		Acidification	Ozone formation	Eutrophication		Ecotoxicity		
		Freshwater ecosystems	Terrestrial ecosystems	Terrestrial	Terrestrial ecosystems	Freshwater	Marine	Freshwater	Marine	Terrestrial
Energy sector	21,982.66	0.31	11,267.85	8027.21	2674.61	4.82	0.08	0.39	0.04	7.37
Mineral industry	3356.92	0.05	1880.22	792.57	681.39	0.47	0.01	0.71	0.03	1.46
Production and processing of metals	2413.07	0.04	1327.94	808.96	261.52	0.53	0.04	0.47	0.06	13.50
Waste and waste-water management	2137.43	0.04	1431.85	215.19	154.81	329.53	2.61	2.73	0.13	0.53
Chemical industry	1748.85	0.03	1000.73	485.51	251.19	9.84	0.17	0.32	0.02	1.06
Intensive livestock production and aquaculture	1307.09	0.00	125.00	1096.47	12.47	72.40	0.27	0.43	0.02	0.03
Paper and wood production and processing	1043.10	0.02	666.78	174.63	192.72	8.22	0.04	0.33	0.02	0.33
Animal and vegetable products from the food and beverage sector	185.92	0.00	73.12	66.43	37.52	8.77	0.02	0.02	0.00	0.05
Unspecied activities	176.61	0.00	70.35	79.27	26.43	0.40	0.01	0.01	0.00	0.14
Other activities	83.10	0.00	15.49	12.71	54.72	0.08	0.00	0.00	0.00	0.10
Total	34,434.75	0.49	17,859.32	11,758.94	4347.38	435.06	3.26	5.40	0.32	24.58

Source: Amalur EIS

**Fig. 3:**
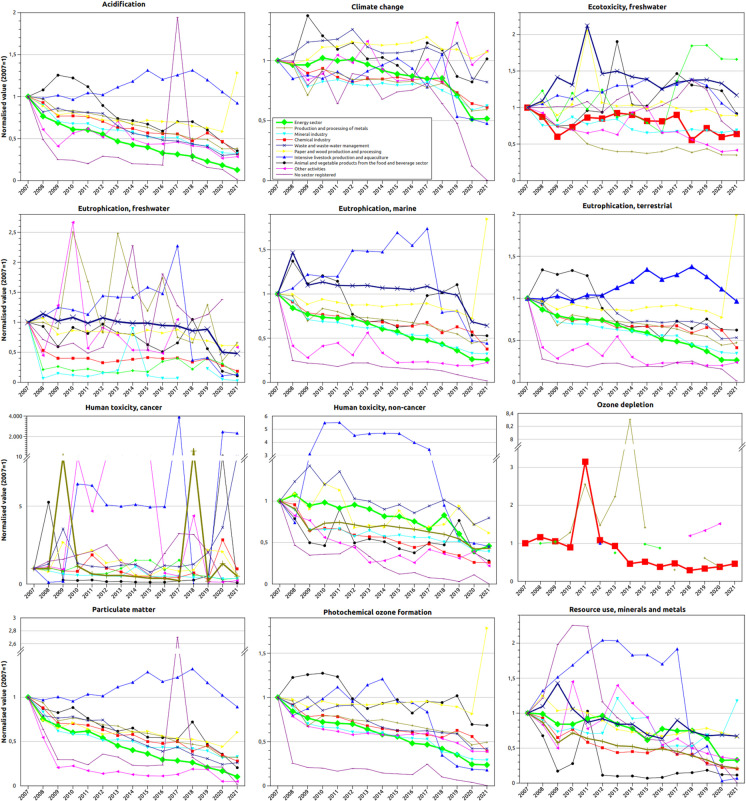
Evolution of EF 3.0 impacts by activity sector (2007–2021). Years refer to years of pollutant emissions. Source: Amalur EIS

Finally, Fig. 3 shows the 2007 to 2021 evolution of the impact of the nine activity sectors considered in the E-PRTR (plus those not specifically recorded), for each of the 12 main impact categories of the EF 3.0 LCIA method. The annual impacts in each sector have been normalised to the impact of each sector in 2007, except in the *ozone depletion* category, where normalisation is carried out with respect to the first annual data recorded. The vertical axes of the graphs for *human toxicities* (*cancer* and *non-cancer*), *ozone depletion* and *particulate matter* are interrupted to facilitate visualisation. The thicker lines correspond to the sectors whose impact amounts to more than 50% of the total. There is a general decrease in EI, although this depends on the specific activity sectors and the impacts considered in each case.

The graphs in Fig. 3 provide substantial trend-level insights, generally indicating a decrease in impacts over time. The energy sector stands out as the most significant in impact terms, accounting for the largest relative contribution in 8 of the 12 impact categories analysed. Despite its prominence, this sector exhibits a consistent downward trend across several impact categories, including *acidification* (− 87.4% from 2007 to 2021), *climate change* (− 48.4%), *eutrophication* (*freshwater* (− 86.4%), *marine* (− 74.6%) and *terrestrial* (− 73.7%)), *human toxicity (non-cancer)* (− 54.1%), *particulate matter* (− 89.8%), *photochemical ozone formation* (− 76.3%) and *resource use (minerals and metals)* (− 67.1%). Other prominent sectors, such as production and processing of metals, mineral industry and chemical industry, also show declining trends in impact generation. However, there are notable exceptions. For instance, the energy sector shows increased impacts in *freshwater ecotoxicity* (+ 66.0%), while the paper and wood production and processing sector shows a moderate rise (+ 7.2%) in *climate change* impacts. Similarly, the intensive livestock production and aquaculture sector and the waste and waste-water management sector exhibit very significant but probably punctual increases in *human toxicity (cancer)*.

The Supplementary Material includes, as an example, two views of the data provided by Amalur EIS. Figure SM7 shows “Aggregated impacts of the activity sectors in the EF 3.0 impact categories”, while Figure SM8 shows “Temporal evolution of the impact of the intensive livestock production sector in the eutrophication terrestrial category for the whole period 2007–2021”.

### Findings related to geographical scope

A geographical analysis using Amalur EIS, for the EF 3.0 LCIA method in its various impact categories, yields the data shown in the annexed Table SM5 “EF 3.0 top 10 facilities” and Table SM6 “EF 3.0 top 10 regions”, provided in the Supplementary Material.

The top ten facilities in each impact category account for between 6 and 16% of the total E-PRTR impact, except for *freshwater ecotoxicity* (32.8%; with seven facilities accounting for between 2.7 and 5.9%), *human toxicity (cancer)* (95.6%; a single Bulgarian facility—Stam Treiding AD—located in the region of Югoзaпaдeн accounting for 86% of total impact) and *ozone depletion* (adding up to 92.3%; with four facilities accounting for between 12 and 24%; along with three facilities in France exceeding 50% of the E-PRTR total impact). Except for these categories, no facility exceeds 5% of total impacts. In relation to regions, the combined impacts of the top ten NUTS 2 regions exceed 15.9% of total E-PRTR impacts in all categories (between 15.9 and 29.5%), except, once again, in *freshwater ecotoxicity* (55%; four regions in Germany account for 25.4%), *human toxicity (cancer)* (95.6%; dominated by the above-mentioned Bulgarian facility) and *ozone depletion* (92.3%; four regions in France exceeding 54% of the total). At this point, we must mention that the case of the Bulgarian Stam Treiding AD facility is included as a data referral error in Annex 4 of (European Environmental Agency, 2024), which recommends the elimination of said record from the table of releases contained in the E-PRTR v18 database. The only record of this facility in the E-PRTR is a 1000.92 kg PCDD + PCDF release to air in 2018, which is obviously erroneous and should be fixed in future updates of the E-PRTR database. It is also necessary to point out that the Amalur EIS development team has not fixed any release reporting error in the E-PRTR v18 database, as this type of corrective action is outside the scope of the present research.

When cross-checking the most pollutant regions and facilities, in 5 of the 12 categories in the EF 3.0 LCIA method, the facility with the highest impact is located in the region with the highest impact (*human toxicities [cancer, non-cancer]*, *acidification*, *ozone depletion* and *resource use*). Düsseldorf in Germany is the top-impact region in four categories (*climate change*, *freshwater ecotoxicity*, *human toxicity [non-cancer]*, *photochemical ozone formation*); in another two, it is the Югoзaпaдeн region, located in Bulgaria (*human toxicity [cancer]*, *acidification*) and two others are regions in Spain (*eutrophication [marine*, *terrestrial]*, Andalucía and Castilla y León, respectively; Figure SM5 shows the significance of the impact of the intensive livestock production sector in the different Spanish regions in the *eutrophication [terrestrial]* category); the rest are located in Norway (*eutrophication [freshwater]*), Serbia (*particulate matter*), France (*ozone depletion*) and Poland (*resource use [minerals and metals]*).

In the Supplementary Material, Table SM7 “EF 3.0 ranking of countries” and Table SM8 “EF 3.0 ranking of NUTS 2 regions” rank 32 countries and 306 NUTS 2 regions, respectively, that are covered by the E-PRTR. Countries and NUTS 2 regions are ranked by total impact after applying the normalisation and weighting steps provided by the EF 3.0 LCIA method. Results are provided as normalised points and as percentages of the total impact for the whole E-PRTR region. Table SM7 shows that only German facilities generate 20.25% of the total impacts, while, taken together, those from the UK (10.45%), Poland (9.65%), Spain (6.97%) and Italy (6.70%) generate 33.76%, with these five countries together accounting for more than 50% of total impacts. If Bulgaria (5.98%), France (5.74%), the Netherlands (4.98%) and Czechia (4.40%) are also taken into account, the impact of these nine countries exceeds 75% of the total. Our analysis of Table SM8 concludes that the 12 regions with the highest aggregate impact for the whole period 2007–2021 are Югoзaпaдeн (Bulgaria, 3.92%; dominated by only two facilities, one of which is clearly erroneous), Düsseldorf (Germany, 3.55%), Köln (Germany, 2.93%), Brandenburg (Germany, 2.45%), Śląskie (Poland, 2.18%), Severozápad (Czechia, 2.01%), Łódzkie (Poland, 1.60%), Zuid-Holland (Netherlands, 1.59%), North Yorkshire (UK, 1.46%), Югoизтoчeн (Bulgaria, 1.39%), Derbyshire and Nottinghamshire (UK, 1.29%) and Sud-Vest Oltenia (Romania, 1.23%), with a combined share of more than 25%. It is noteworthy that these regions alone score higher than entire countries such as Ireland, Slovakia, Hungary, Estonia or Austria. Table SM9 shows that in those 12 NUTS 2 regions with the greatest impacts, the *climate change* category presents a contribution above the average, except in three cases: in Югoзaпaдeн (Bulgaria), *human toxicity (cancer)* stands out (92%) due to an erroneous reporting, and in Югoизтoчeн (Bulgaria) and Sud-Vest Oltenia (Romania), *climate change* drops to 46% while impacts in *acidification* and *particulate matter* are greater than 20% (averages are 7.5% and 6.4%, respectively). In addition, two Extra-Regio NUTS 2 areas present significant impacts on the sea. These areas are associated with the UK, ranking 54th out of 306 with an impact of 0.55%, and with Norway, ranking 94th out of 306 with an impact of 0.35%. Their impacts are higher than those of countries such as Slovenia (0.36%) and Switzerland (0.31%), respectively.

Table SM9 “EF 3.0 normalised and weighted impacts for countries and regions by impact category” shows that the dominant impact category is *climate change* (68.6% of the total in all E-PRTR), but with differences depending on the country. Among those with the highest total impacts, the Netherlands (88.8%), Germany (83.0%) and Czechia (79.4%) have an above-average impact in the *climate change* category. Others are below the average: Spain (55.8% in *climate change*) presents greater-than-average impacts in *acidification* (12.2% vs 7.5%) and *eutrophications* (12% vs 6.3%). In Bulgaria, *human toxicity (cancer)* impact is disproportionately high (60.5% vs 4.2%) due to an erroneous reporting.

In addition, the total sectoral impacts for the whole period 2007–2021 are shown in Table SM10 “EF 3.0 normalised and weighted impacts for countries and regions by activity sector”. As noted before, the energy sector generates the highest total impact (59.5%), with a wide gap over the rest, as the following four sectors together account for less than 40% of the total impact: production and processing of metals (11.4%), mineral industry (9.3%), waste and waste-water management (6.6%) and chemical industry (6.1%). The weight of the energy sector is particularly relevant in some countries, such as Estonia (93.7%), Serbia (87.2%), Greece (84.1%), Czech Republic (75.2%), Denmark (69.3%), Netherlands (68.7%), Cyprus (68.3%), Poland (67.7%), Germany (67.2%) and the UK (66.7%), where it exceeds the average value (59.5%). In these countries, the rest of the sectors in general do not differ much from the average values, with the exception of the mineral industry in Cyprus (25.8%), Denmark (14.9%) and Poland (12.7%), and the waste sector in the UK (13.7% vs 6.6%). Other countries, however, show a different sectoral behaviour. In France, where nuclear power is very significant, all other important sectors exceed the average values: production and processing of metals (16.2%), mineral industry (12.6%), waste and waste-water management (11.9%) and chemical industry (12.6%). On the other hand, in some other countries, the impacts derived from the intensive livestock production and aquaculture sector show values well above the average value (2.7%), as is the case in Norway (11.9%), Portugal (8.1%), Spain (7.9%), Romania (7.3%) and Italy (4%).

## Discussion and conclusions

Amalur EIS allows us to analyse the environmental impacts derived from the emissions registered in the E-PRTR for the main industrial facilities in Europe. The use of the EF 3.0 LCIA method allows for the quantification of environmental impacts in a variety of impact categories—namely, *climate change*, *ecotoxicity*, *human toxicities*, *photochemical ozone formation*, *acidification*, *eutrophications*, *particulate matter*, *ozone depletion* and *resource use (minerals and metals*)—of a large number of facilities in different activity sectors, countries and regions. In addition, other LCIA methods can also be applied within the software, which may help to uncover divergences and complementarities between the different methods. While TRACI is promoted by the US Environmental Protection Agency, EF is promoted by the European Commission and provides a different geographic scope. Many methods incorporate the GWP100 metric for global warming, but IPCC methods also consider other metrics and time horizons. Some methods are more comprehensive (CML-IA, ReCiPe, EF, TRACI), while others are more specific, like IPCC (focused on *climate change*) and USEtox (focused on *ecotoxicity* and *human toxicities*). While EPS is an endpoint method assessing economic damage, ReCiPe considers the areas of protection human health, ecosystem quality and natural resources at endpoint level. Combining insights from different LCIA methods can provide a broader understanding of environmental impacts. Results from one method can help validate findings from another, strengthening confidence in the results or highlighting areas where further analysis may be needed if results diverge.

The environmental impacts from all E-PRTR facilities calculated with the EF 3.0 method for the period 2007–2021 are dominated by four sectors of activity. The energy sector presents the highest impacts in the categories of *climate change*, *eutrophications (marine*, *terrestrial)*, *human toxicity (non-cancer)*, *particulate matter* and *photochemical ozone formation*. The sector of production and processing of metals takes first place in *human toxicity (cancer)* and *resource use (minerals and metals)*. Meanwhile, the mineral industry sector leads in the categories of *ecotoxicity* and *ozone depletion*. The waste sector leads in the *eutrophication (freshwater)* category. It is worth noting that the intensive livestock production and aquaculture sector generates between 16 and 20% in two of the three *eutrophication* categories and between 10 and 11% in *acidification* and *particulate matter* (see Table 2).

Activity sectors generally show a decrease in environmental impacts over time. This downward trend is especially evident for the energy sector, which is the most significant contributor in 8 of the 12 main impact categories of the EF method. As a result of the decarbonisation policies promoted by the European Union, the energy sector shows substantial impact reductions from 2007 to 2021 that, for example, reach 48.8% in *climate change*, 87.4% in *acidification* and 89.8% in *particulate matter*. Other notable sectors, such as production and processing of metals and the mineral and chemical industries, also exhibit declining trends in impact generation.

The normalisation and weighting steps provided by EF 3.0 aggregate in a single indicator the total impact of E-PRTR facilities for all categories. This allows us to conclude that facilities in the five most polluting countries contribute more than 50% of the total impact (Germany, 20.25%; UK, 10.45%; Poland, 9.65%; Spain, 6.97% and Italy, 6.70%). Leaving aside the region of Югoзaпaпaдeн (Bulgaria), where a facility with a serious data reporting error is located, the regions that account for more than 2% of the total impact are as follows: Düsseldorf (Germany), with 3.55% of the total impact and exceeding average values in three impact categories (*climate change*, 81.5% vs 68.6%; *ecotoxicity [freshwater]*, 4.1% vs 1.5%; *human toxicity [non-cancer]*, 2% vs 1.2%); Köln (Germany), 2.93%; Brandenburg (Germany), 2.45%; Śląskie (Poland), 2.18% and Severozápad (Czechia), 2.01%. In these five regions, the energy sector has by far the highest impact, all of them showing values above 50% going above average (59.5%) in four of them (Düsseldorf, 66.53%; Köln, 87.54%; Brandenburg, 87.92%; Severozápad, 83.45%). The impacts of the production and processing of metal sector are significant in Düsseldorf (20.61% vs 11.4% of the average value), while in the Śląskie region, the mineral industry (18% vs 9.3%) and intensive livestock production and aquaculture (7.37% vs 2.7%) stand out.

Without a doubt, these impacts are related to the economic structure of the regions. For example, the German regions of Düsseldorf and Köln have a high level of economic development, with per capita incomes in 2022 of 119% and 121% of the EU27 average, respectively (Eurostat, 2024). Furthermore, both are located in the state of North Rhine-Westphalia, in the west of the country, known for its high industrialisation. It is also a state historically linked to coal mining, along with other regions in the east of the country, such as Brandenburg. In fact, Germany is a country heavily dependent on fossil fuels, with 77.6% of primary energy consumption coming from fossil sources in 2023 (AG Energiebilanzen – Working Group on Energy Balances, 2024). Although coal has a decreasing relative weight, it still accounts for 17% of primary energy consumption, while industry accounts for 28% (ibid.). In fact, the state of North Rhine-Westphalia aims to be a national leader in the reduction of coal use by 2029 (Ministry of Economic Affairs, 2024). The performance of these regions in any case reflects the fact that the energy sector is strongly linked to climate change, as also reported in other works (Erhart & Erhart, 2023). In fact, the most important finding of our analysis is that the energy sector is the largest generator of impacts (59.5%), among which the *climate change* category is the largest (68.6%) contributor to the total impact.

The analysis of results has also validated the relevance and usefulness of Amalur EIS, consolidating this EIS as a comprehensive, multidimensional and user-friendly tool. Amalur EIS provides wide geographical coverage, as it includes the European countries reporting emissions to E-PRTR, and also provides disaggregated information at regional level and by exact location of the installations. Moreover, Amalur EIS’ LCIA methods provide extensive coverage of reported emissions, reaching 92% effectiveness of pollutants registered in the E-PRTR. It currently contains data for the period 2007–2021 but may be updated in the future as the European Industrial Emissions portal makes more E-PRTR data available. The Amalur EIS website (https://www.amalur-eis.eus/) will make its data and derived environmental impacts easily accessible to everyone.

Unlike Amalur EIS, other software does not include LCIA methods for EI quantification (Dios et al., 2014; Overberg et al., 2023), and where LCIA methods are included (Erhart & Erhart, 2023; Sörme et al., 2016) they are more limited than Amalur EIS, which offers 18 LCIA methods (31 in fact, if we take into account the different versions of several of them). Applying multiple methods allows for cross-validation, enhancing the robustness of results and identifying areas that may require additional analysis. It also offers the opportunity to estimate numerous environmental impacts beyond the well-known and extensively used *climate change* impact category. Although this category is truly relevant (68.6% of total EF 3.0 normalised and weighted impacts of the whole E-PRTR) and the most prominent on the international environmental agenda today, tools such as Amalur EIS underline the relevance of others, such as *acidification* (7.5% of total impact), *particulate matter* (6.4%), *eutrophications* (6.3%) and *human toxicities* (5.4%). Many other impacts resulting from human activity are thus highlighted, the detailed information of which is very valuable for a multidimensional and integrated interpretation of sustainability. From a decision-making perspective, it can ultimately help to raise the importance of some environmental impacts that may not be as high on the public agenda.

The E-PRTR database is a well-founded information source, which has been improved over the years. However, the Amalur EIS construction process has revealed some opportunities for improvement. As noted, when analysing top releases, reporting data quality errors have been detected. This is consistent with other authors’ conclusions (Dios et al., 2014; Erhart & Erhart, 2023; European Environmental Agency, 2024; Fikru, 2011) so correcting these errors and limiting future ones as much as possible would contribute to a more consistent E-PRTR database.

Beyond the data provided by E-PRTR, the EI information provided by Amalur EIS is relevant, both from the point of view of access to public information and for public decision-making. The importance of public access to environmental information was recognised by the United Nations Conference on Environment and Development (UNCED) in 1992 (United Nations, 1992) through Agenda 21 (UNCED, 1992) and Principle 10, which emphasises environmental information and public participation in decision-making. Again, we must underline the importance of E-PRTR in terms of public access to information on environmental pollution, and, in the same vein, the value of knowing the environmental impacts linked to such emissions as a step forward. Amalur EIS may also help strengthen the reputation of companies along the same lines as the E-PRTR does (Cañón-de-Francia et al., 2008; Fikru, 2011). Additionally, Amalur EIS could also be useful in the public policy arena. It would contribute to more effective environmental policy, as it can identify diverse environmental impacts by geographical locations, types of emissions and sectors of industrial activity, among others. As a decision support tool at national or regional level, it also provides complementary information to sectoral environmental information systems (e.g. Cifrian et al., 2015).

Despite the extensive analytical scope of Amalur EIS, it is believed that the software could be complemented in the future to amplify its potential. Actually, the calculation of environmental impacts from diffuse air releases for year 2008 has been already implemented into our EIS software, although results will be analysed in a future publication. The inclusion of both socio-economic and demographic data is also foreseen. This information could consist of indicators such as population density, per capita income, income distribution inequality, industrial value added and industrial employment and productivity by activity sector at the European regional level. Finally, there is also the intention to add geographically located health-related data for cross-checking with impacts provided by Amalur EIS in the area of protection of human health. The ReCiPe 2016 Endpoint (I) method, for example, provides an impact in human health of 2.77 million DALY for the whole E-PRTR record (geographical distributions are also available; total results are shown in Figure SM6, where once again the energy sector is dominant, with 63.0% of total impacts). Furthermore, the EPS 2015dx method incorporates impact categories in relation to specific diseases, such as *asthma cases* (Amalur EIS provides an impact estimation of 280 thousand person·year for the 2007–2021 period derived from the activity of the E-PRTR facilities), *diarrhoea* (impact of 511 thousand person·year) or *YOLL* (years of life lost, with an impact of 23.7 million person·year). Figure SM9 “Activity sector impacts (person·year) in EPS 2015dx categories”, provided in the Supplementary Material, shows activity sector impacts in these and other EPS 2015dx categories. These areas of further improvement do not, however, detract from the functionality and analytical capacity of all data currently available on the Amalur EIS website.

In terms of empirical development and future research, we believe that Amalur EIS may contribute to progress in three areas, at the very least. Firstly, Amalur EIS can contribute to the evaluation of surpassing certain planetary boundary thresholds, in line with specific guidelines provided by the LCA framework (Sala et al., 2016). Thanks to its nature, it could be particularly helpful in the quantification of impacts linked to Novel Entities, a boundary whose “impacts on Earth system as a whole remain largely unstudied” (Richardson et al., 2023). Secondly, it is a useful tool for advancing in the field of environmental justice and ecological distribution conflicts. Amalur EIS’ ability to geographically locate environmental impacts and associate them with certain activity sectors is valuable in this regard. Indeed, in the search for justice, facilities and/or economic activities that cause pollution are opposed by local people and civil society actors for their environmental and human health impacts (Temper et al., 2015). The information provided by Amalur EIS in terms of geographically located environmental impacts could contribute to transparency in such conflicts, as it relates industrial metabolism with impacts generated. Finally, the analyses provided by Amalur EIS can also be complemented with other types of socio-economic and demographic variables at the regional level. This combination of variables offers the opportunity to expand the field of action towards the integration of economic, health and environmental inequalities at the European level. This is a line of work that has been conducted thus far using information from the E-PRTR (Fernández-Navarro et al., 2017; Neier, 2021; Rüttenauer, 2018) and can now be extended with the help of Amalur EIS. In the same vein, other global emission registries could also be easily incorporated into this EIS (e.g. US TRI, Canadian NPRI, Mexican RETC, Australian NPI).

In conclusion, the main aim of Amalur EIS is to make all its data available to the academic community, policymakers, businesses and civil society. Its potential in terms of calculating industrial sectors’ environmental impacts is enormous, which we believe makes it a very valuable tool for the transition towards sustainability, particularly given the challenges faced by Europe at this time.

## Supplementary Information

Below is the link to the electronic supplementary material.

ESM 1 (PNG 1.32 MB)

Supplementary file1 (TIFF 205 KB)

ESM 2 (PNG 2.16 MB)

Supplementary file2 (TIFF 408 KB)

ESM 3 (PNG 2.23 MB)

Supplementary file3 (TIFF 418 KB)

ESM 4 (PNG 1.02 MB)

Supplementary file4 (TIFF 229 KB)

ESM 5 (PNG 2.33 MB)

Supplementary file5 (TIFF 506 KB)

ESM 6 (PNG 926 KB)

Supplementary file6 (TIFF 188 KB)

ESM 7 (PNG 1.42 MB)

Supplementary file7 (TIFF 313 KB)

ESM 8 (PNG 8.14 KB)

Supplementary file8 (TIFF 249 KB)

ESM 9 (PNG 1.28 MB)

Supplementary file9 (TIFF 338 KB)

ESM 10 (PNG 8.55 MB)

Supplementary file10 (TIFF 1468 KB)

Supplementary file11 (XLSX 39 KB)

Supplementary file12 (XLSX 76 KB)

Supplementary file13 (XLSX 19 KB)

Supplementary file14 (XLSX 20 KB)

Supplementary file15 (XLSX 24 KB)

Supplementary file16 (XLSX 39 KB)

Supplementary file17 (XLSX 18 KB)

Supplementary file18 (XLSX 35 KB)

Supplementary file19 (XLSX 143 KB)

Supplementary file20 (XLSX 112 KB)

## Data Availability

E-PRTR data: https://industry.eea.europa.eu/download LCIA data: https://www.openlca.org/lca-data/ Amalur EIS data: https://amalur-eis.eus/

## Footnotes

1 Note: The three possible emission media are *air*, *land* or *water*. In addition, each medium may be associated with different environments: *high/low population density*, *lower/upper stratosphere* or *indoor* for the *air* medium; *forestry*, *agricultural* or *industrial* for the *land* medium and *surface water*, *ocean*, *lake*, *river* or *freshwater* for the *water* medium. openLCA uses the terminology *category/sub-category* to refer to *medium/environment*, in the same way that Erhart and Erhart (2022) use that of *sub-compartment*.
