# Supplementary figures and images for: Amalur EIS: a system for calculating the environmental impacts of industrial sites from E-PRTR records
Source: Environ Monit Assess. 2025 Jan 11;197(2):163. doi: 10.1007/s10661-024-13565-3 (PMC11723853; doi:10.1007/s10661-024-13565-3)

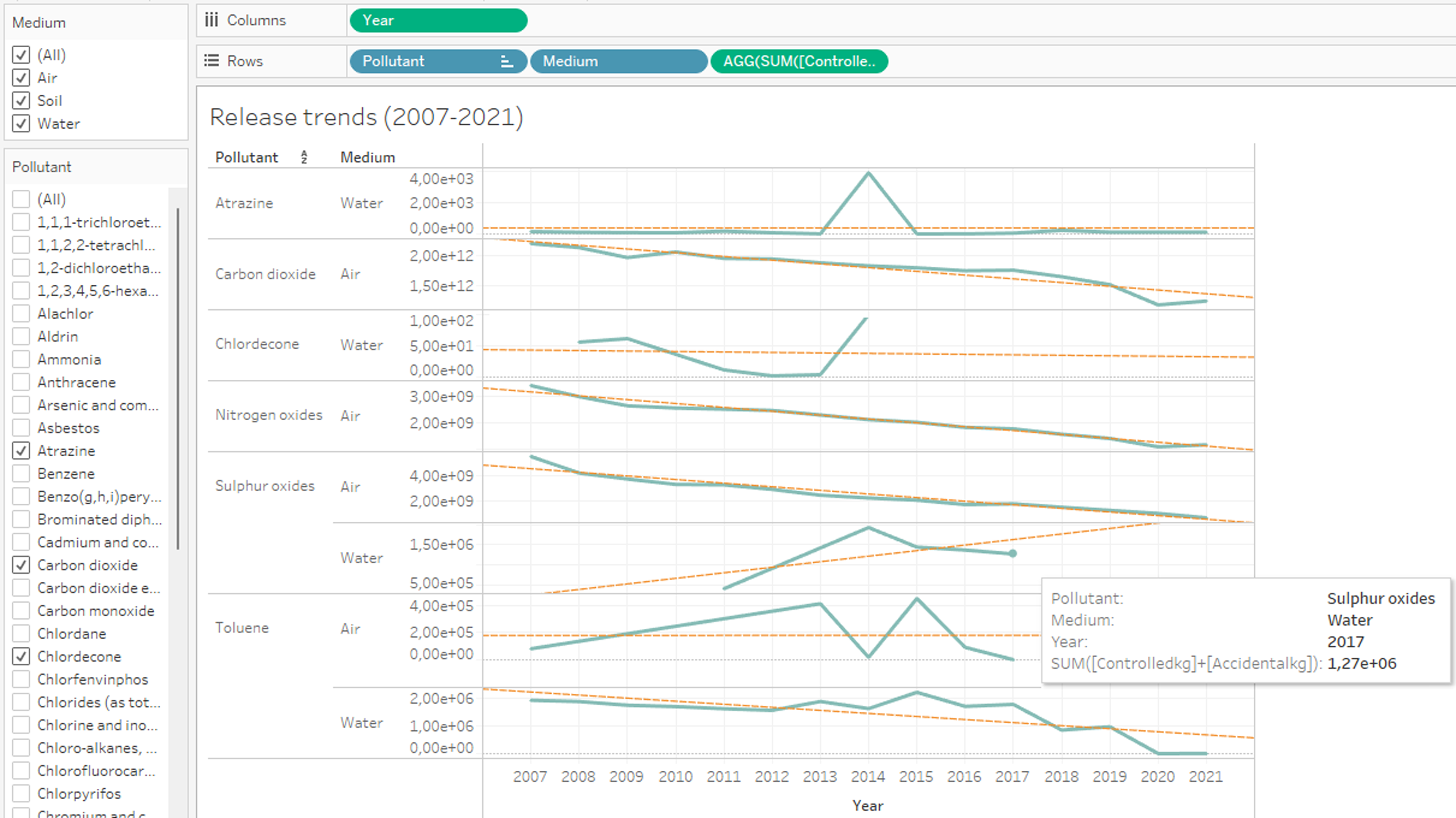

Supplement: Supplementary file 1 — (PNG 1.32 MB) [file 10661_2024_13565_Fig4_ESM.png]

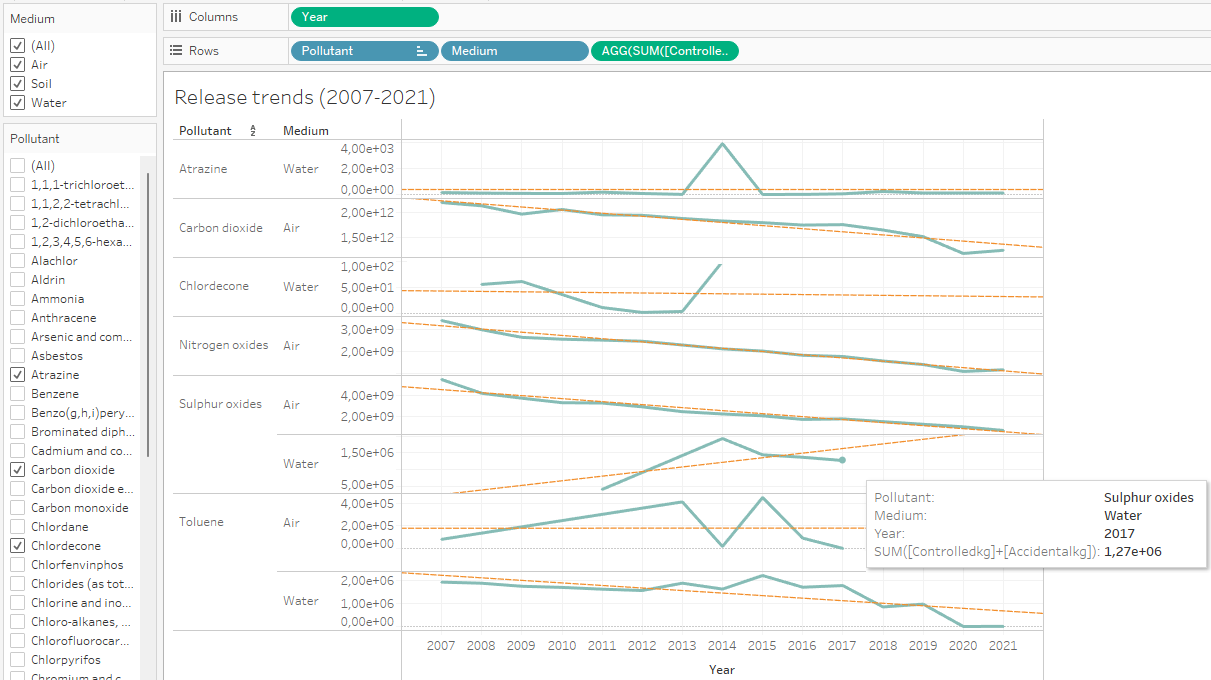

Supplement: Supplementary file 2 — Supplementary file1 (TIFF 205 KB) [file 10661_2024_13565_MOESM1_ESM.tiff]

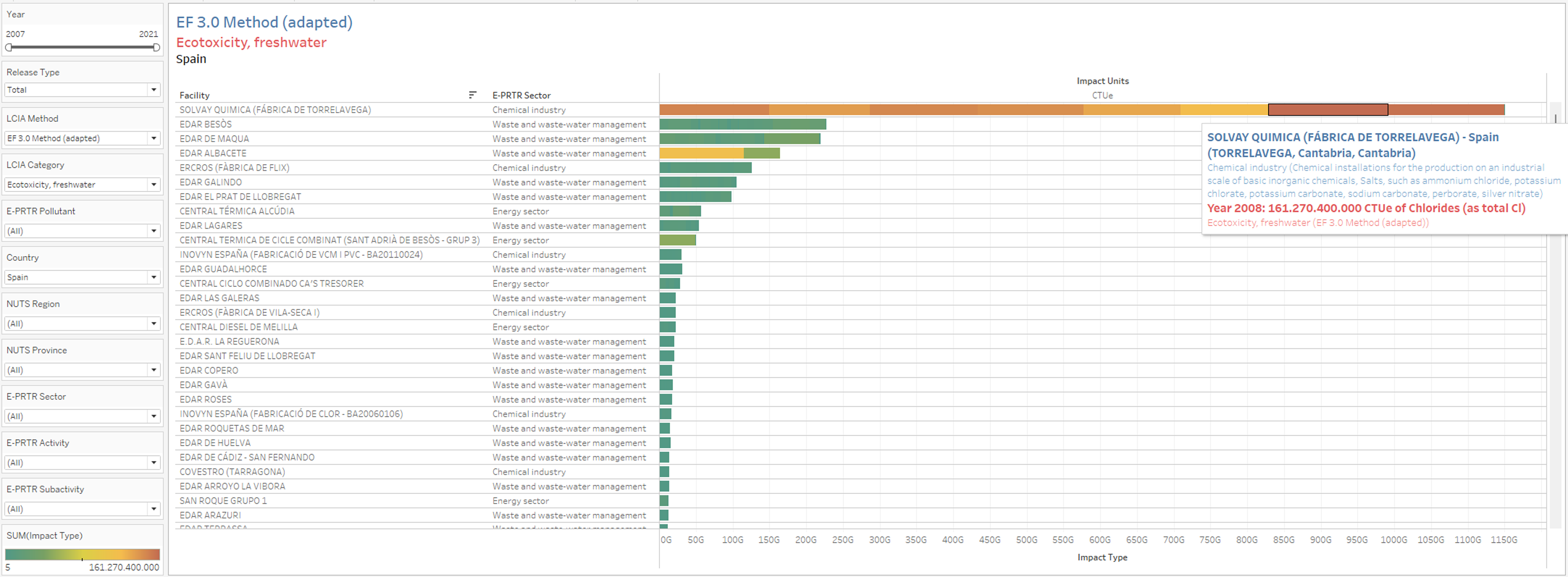

Supplement: Supplementary file 3 — (PNG 2.16 MB) [file 10661_2024_13565_Fig5_ESM.png]

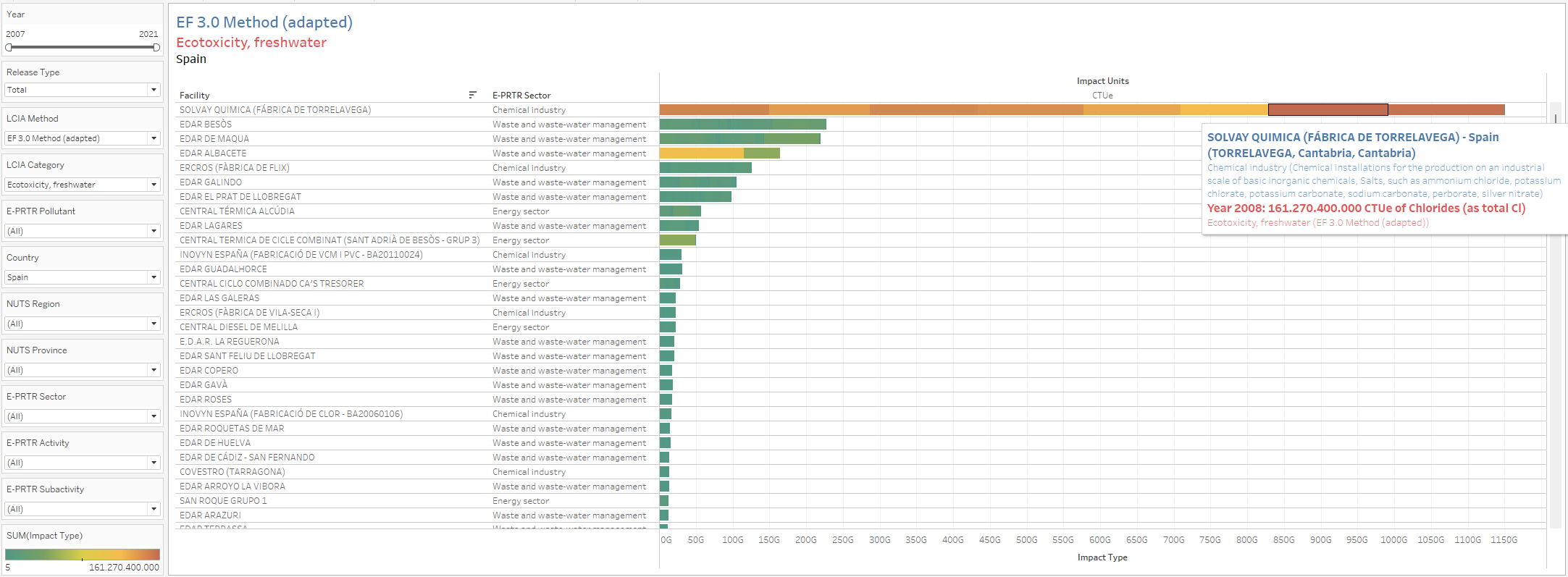

Supplement: Supplementary file 4 — Supplementary file2 (TIFF 408 KB) [file 10661_2024_13565_MOESM2_ESM.tiff]

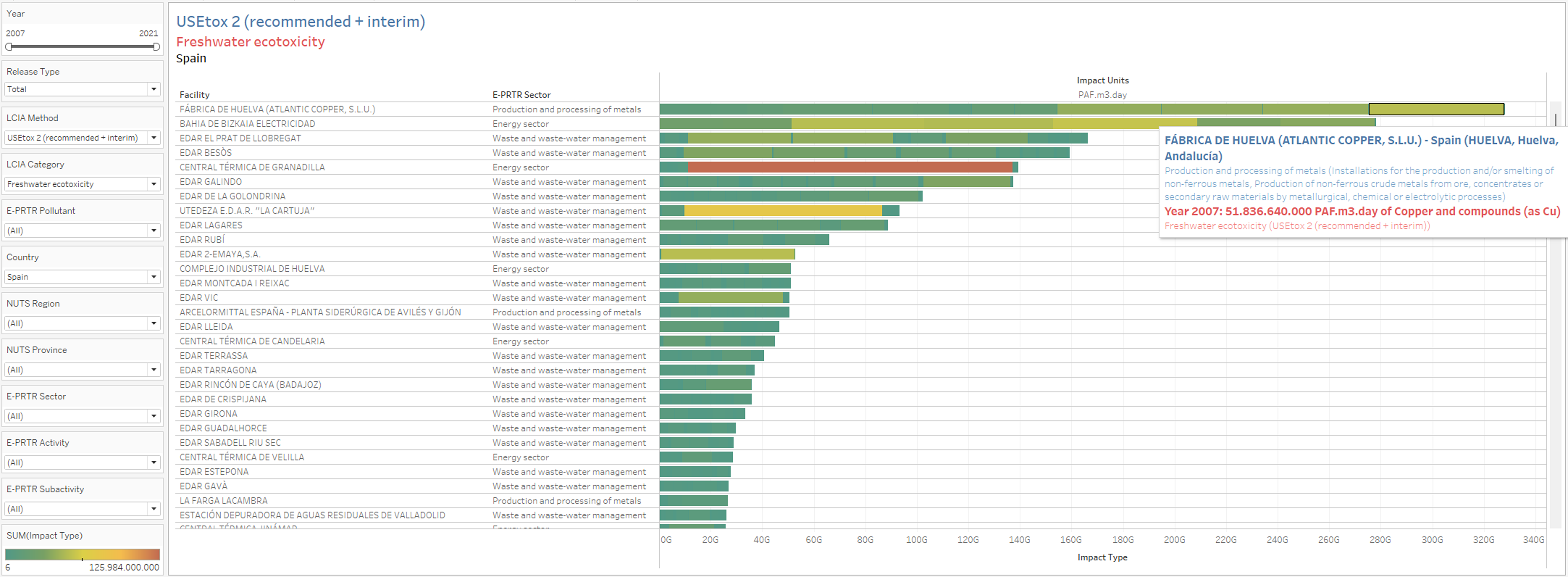

Supplement: Supplementary file 5 — (PNG 2.23 MB) [file 10661_2024_13565_Fig6_ESM.png]

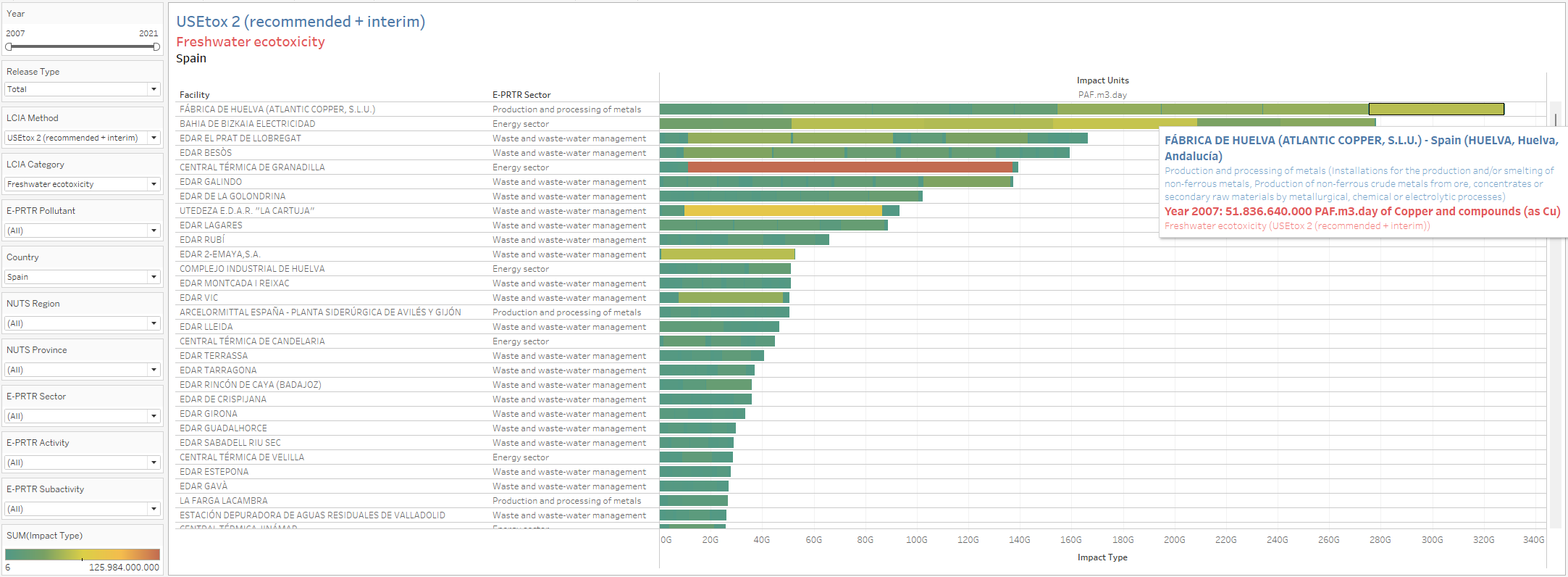

Supplement: Supplementary file 6 — Supplementary file3 (TIFF 418 KB) [file 10661_2024_13565_MOESM3_ESM.tiff]

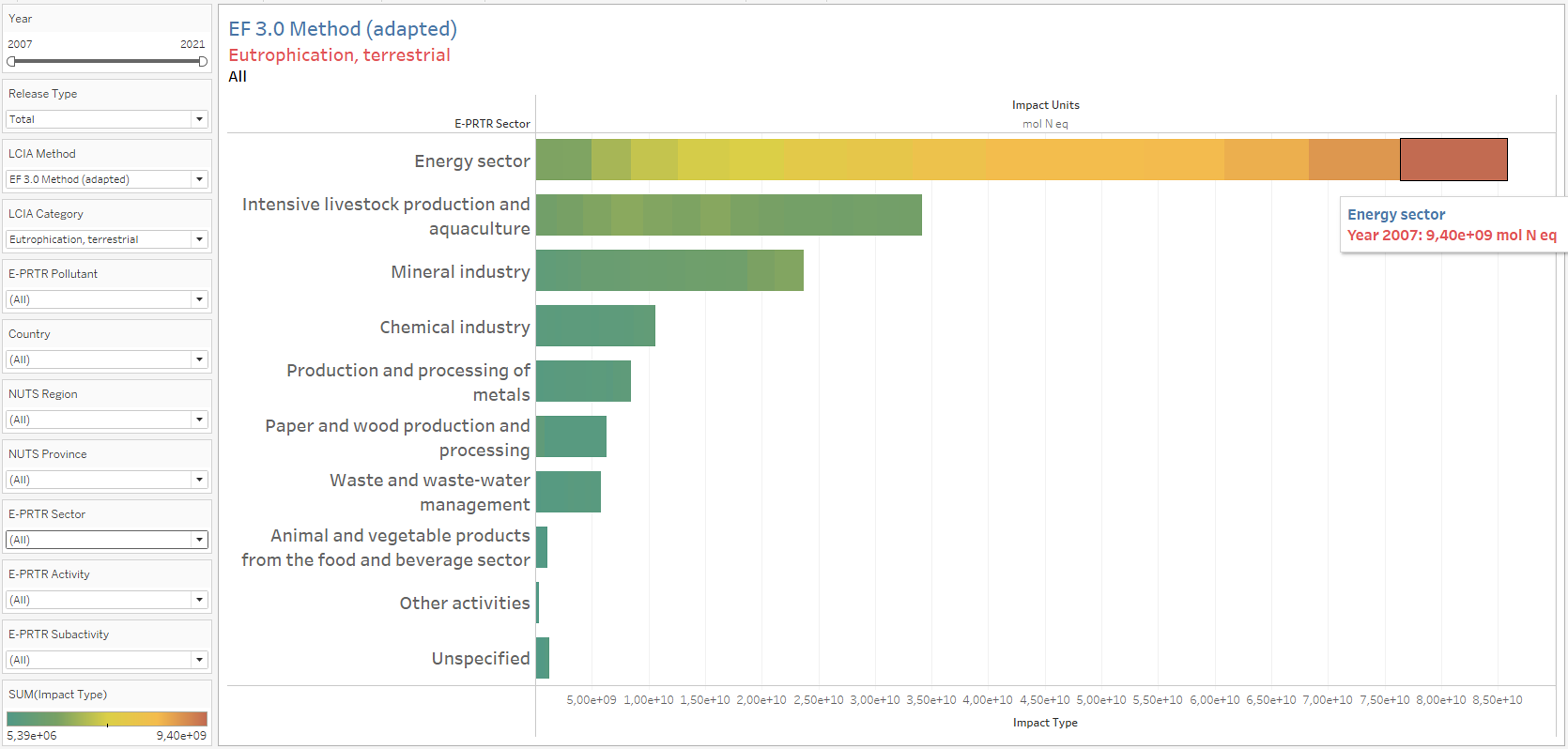

Supplement: Supplementary file 7 — (PNG 1.02 MB) [file 10661_2024_13565_Fig7_ESM.png]

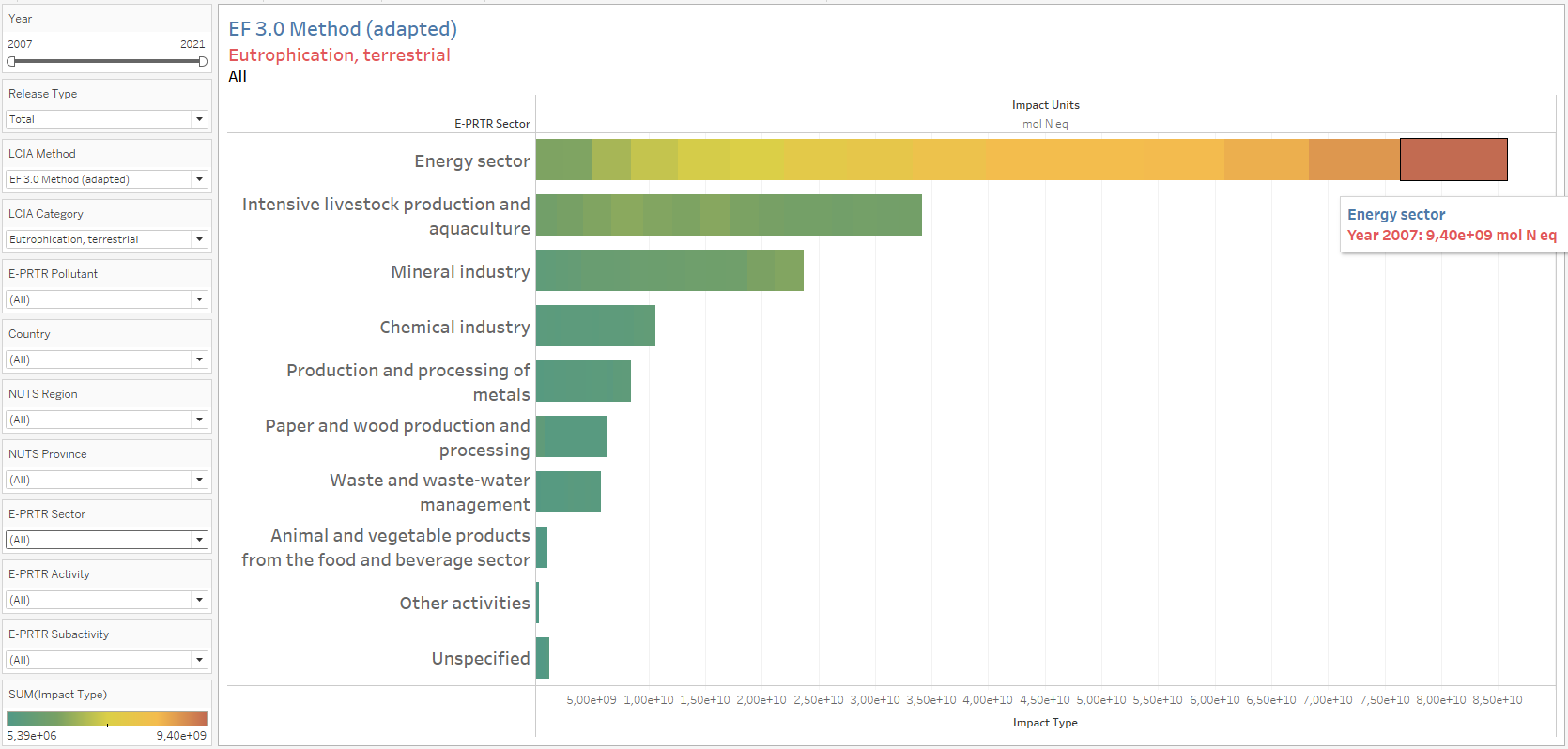

Supplement: Supplementary file 8 — Supplementary file4 (TIFF 229 KB) [file 10661_2024_13565_MOESM4_ESM.tiff]

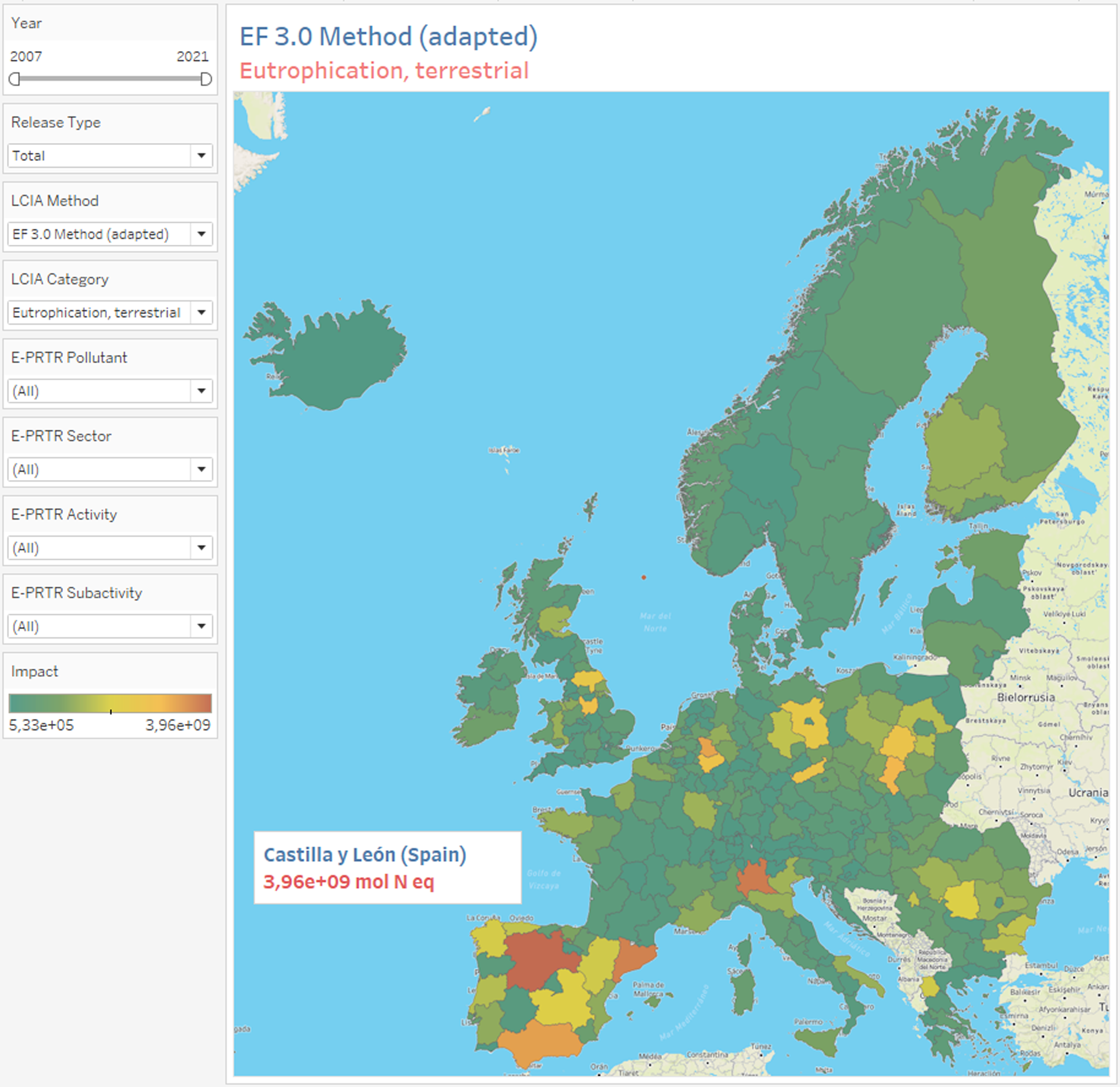

Supplement: Supplementary file 9 — (PNG 2.33 MB) [file 10661_2024_13565_Fig8_ESM.png]

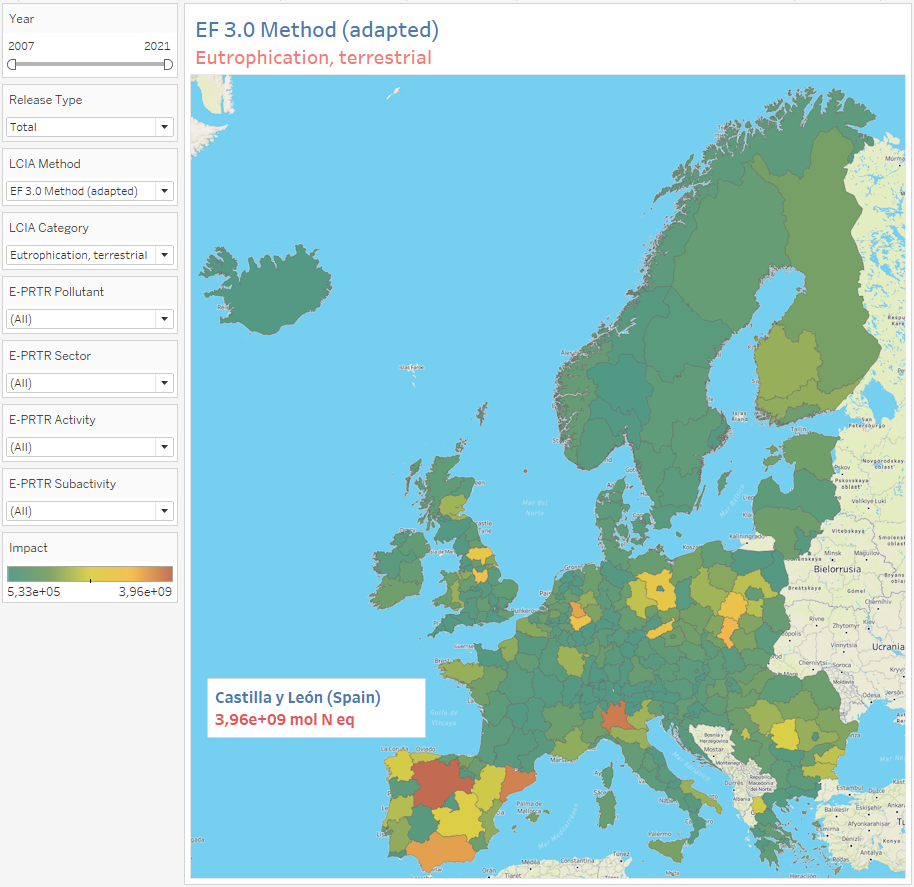

Supplement: Supplementary file 10 — Supplementary file5 (TIFF 506 KB) [file 10661_2024_13565_MOESM5_ESM.tiff]

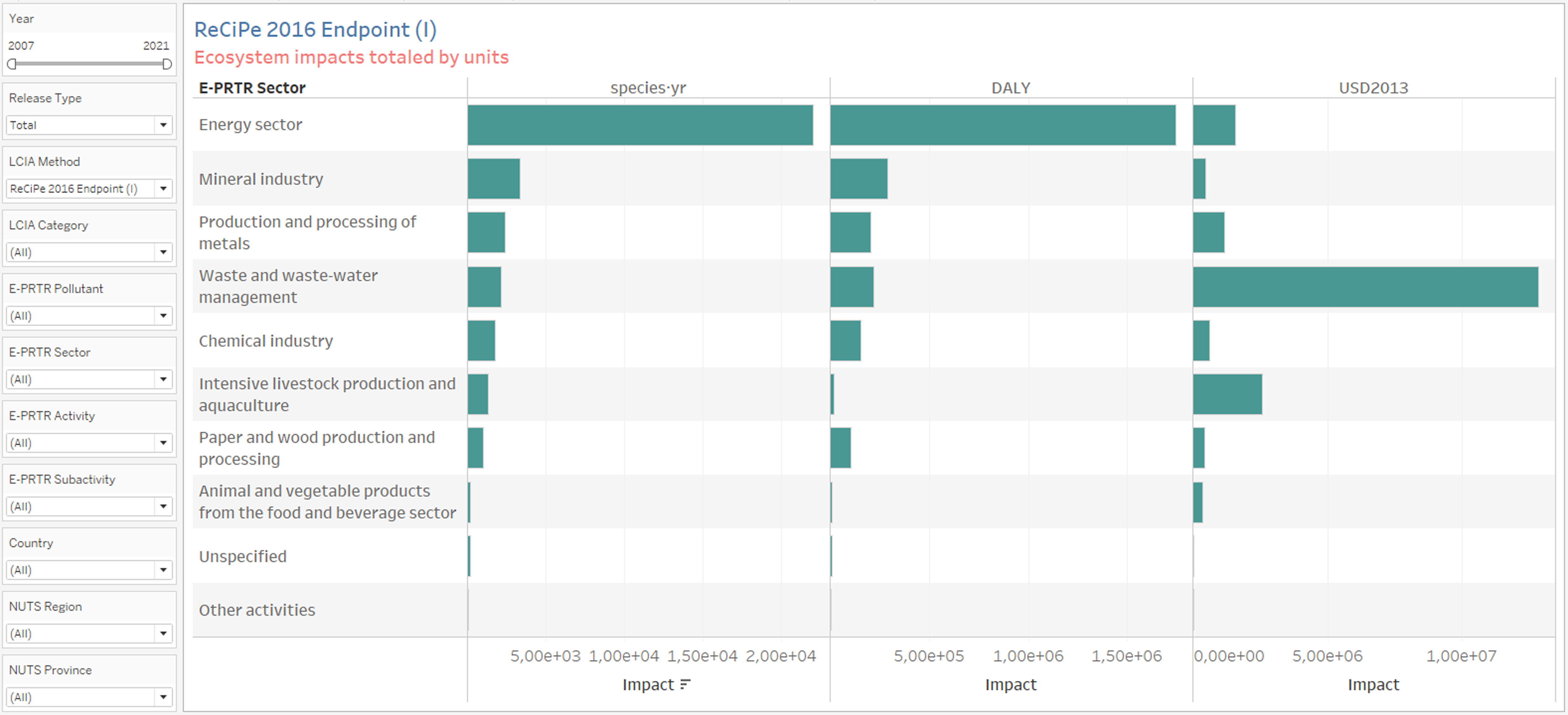

Supplement: Supplementary file 11 — (PNG 926 KB) [file 10661_2024_13565_Fig9_ESM.png]

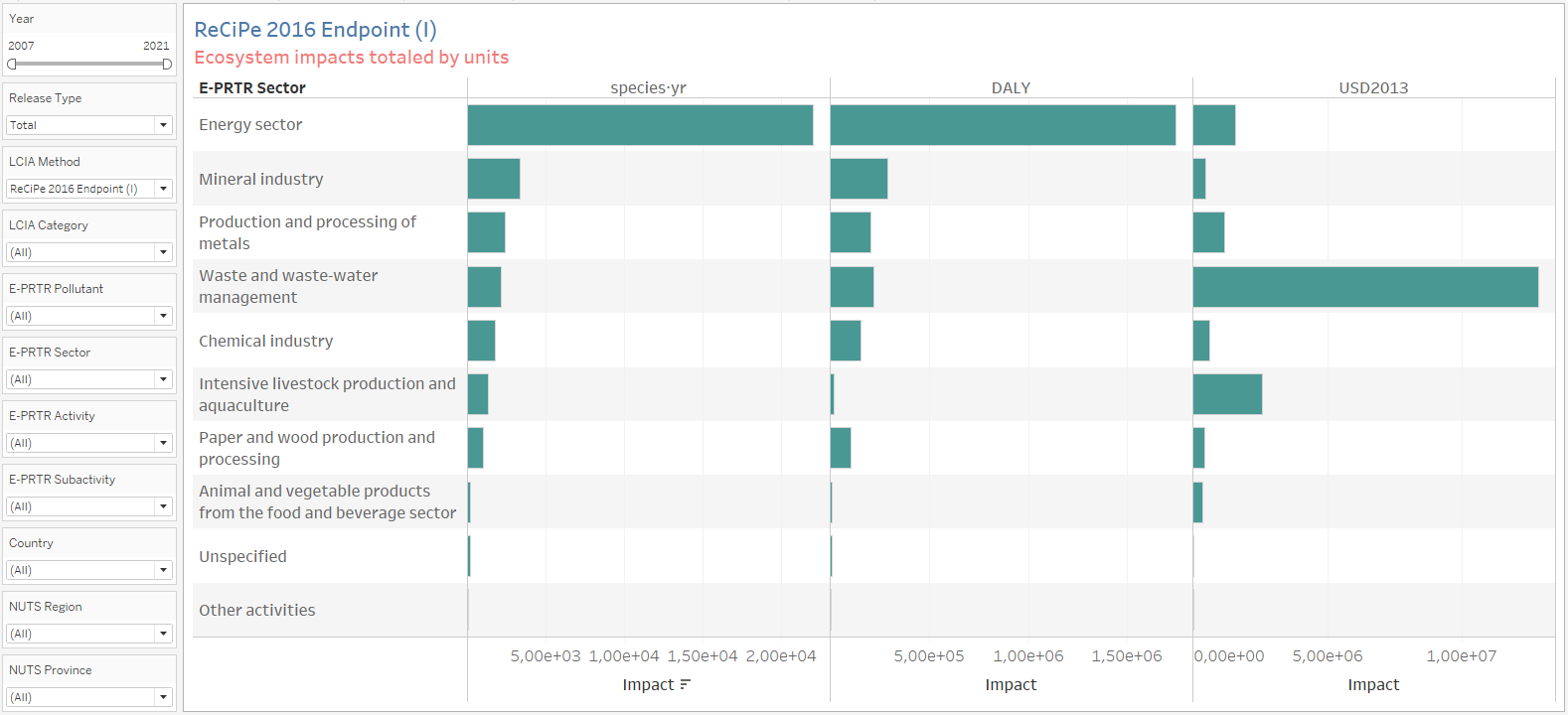

Supplement: Supplementary file 12 — Supplementary file6 (TIFF 188 KB) [file 10661_2024_13565_MOESM6_ESM.tiff]

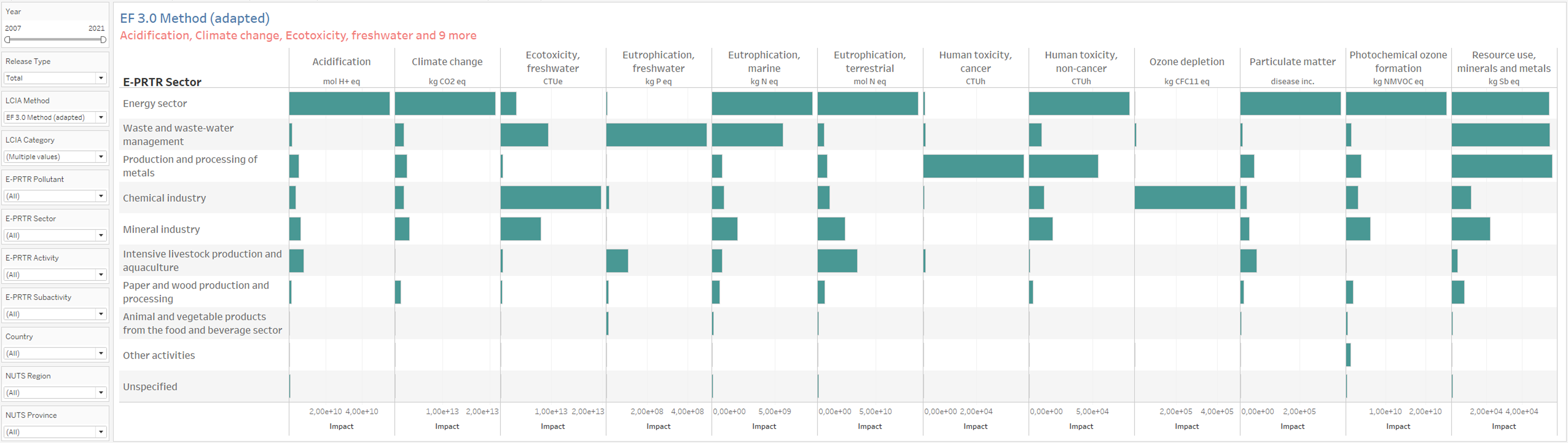

Supplement: Supplementary file 13 — (PNG 1.42 MB) [file 10661_2024_13565_Fig10_ESM.png]

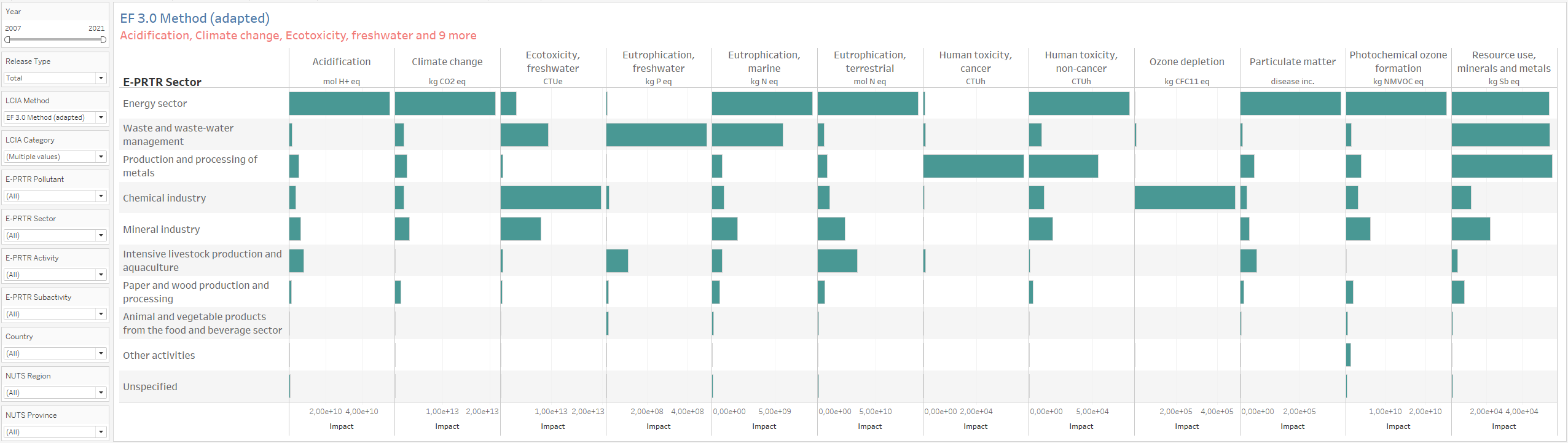

Supplement: Supplementary file 14 — Supplementary file7 (TIFF 313 KB) [file 10661_2024_13565_MOESM7_ESM.tiff]

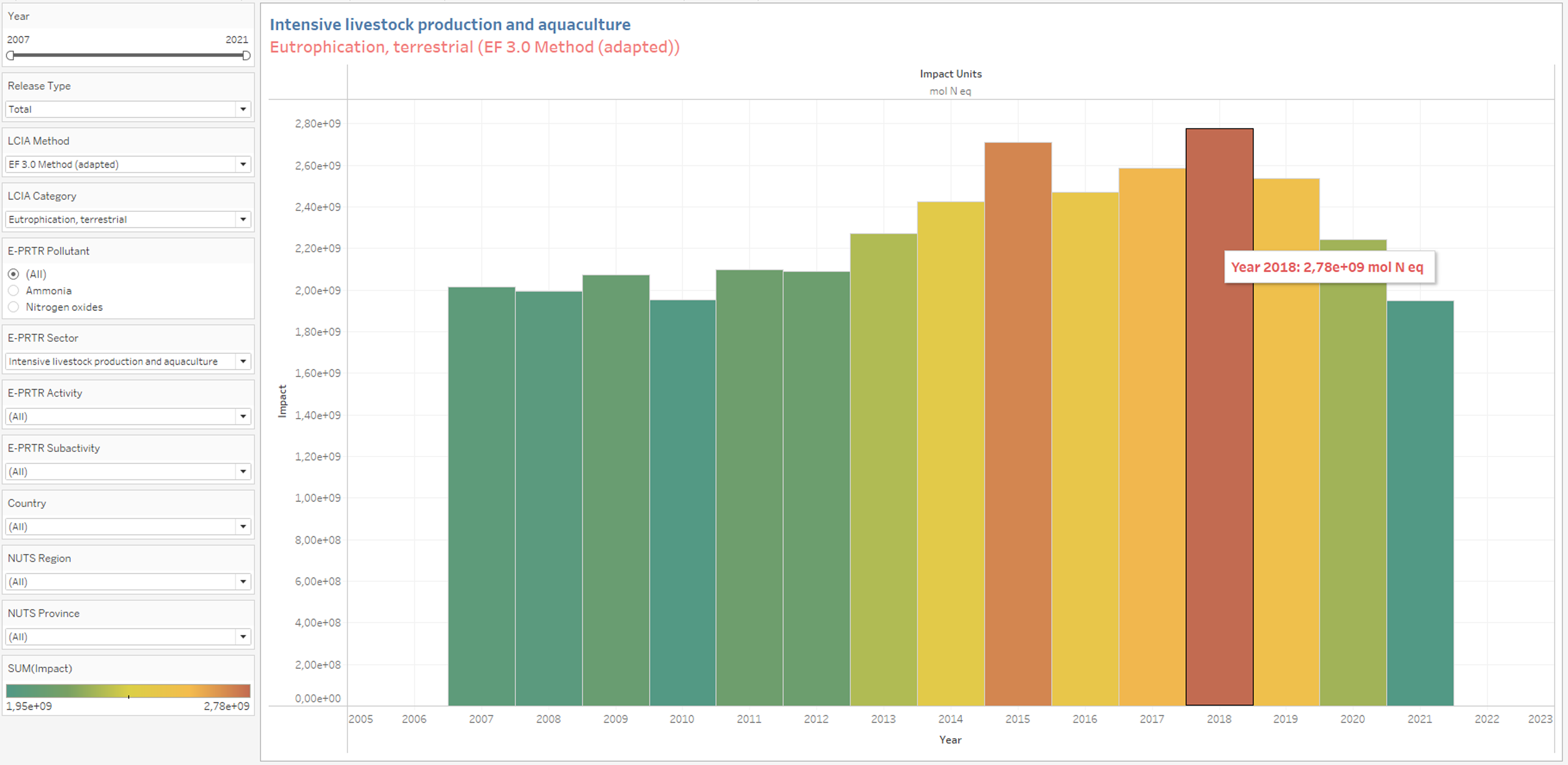

Supplement: Supplementary file 15 — (PNG 8.14 KB) [file 10661_2024_13565_Fig11_ESM.png]

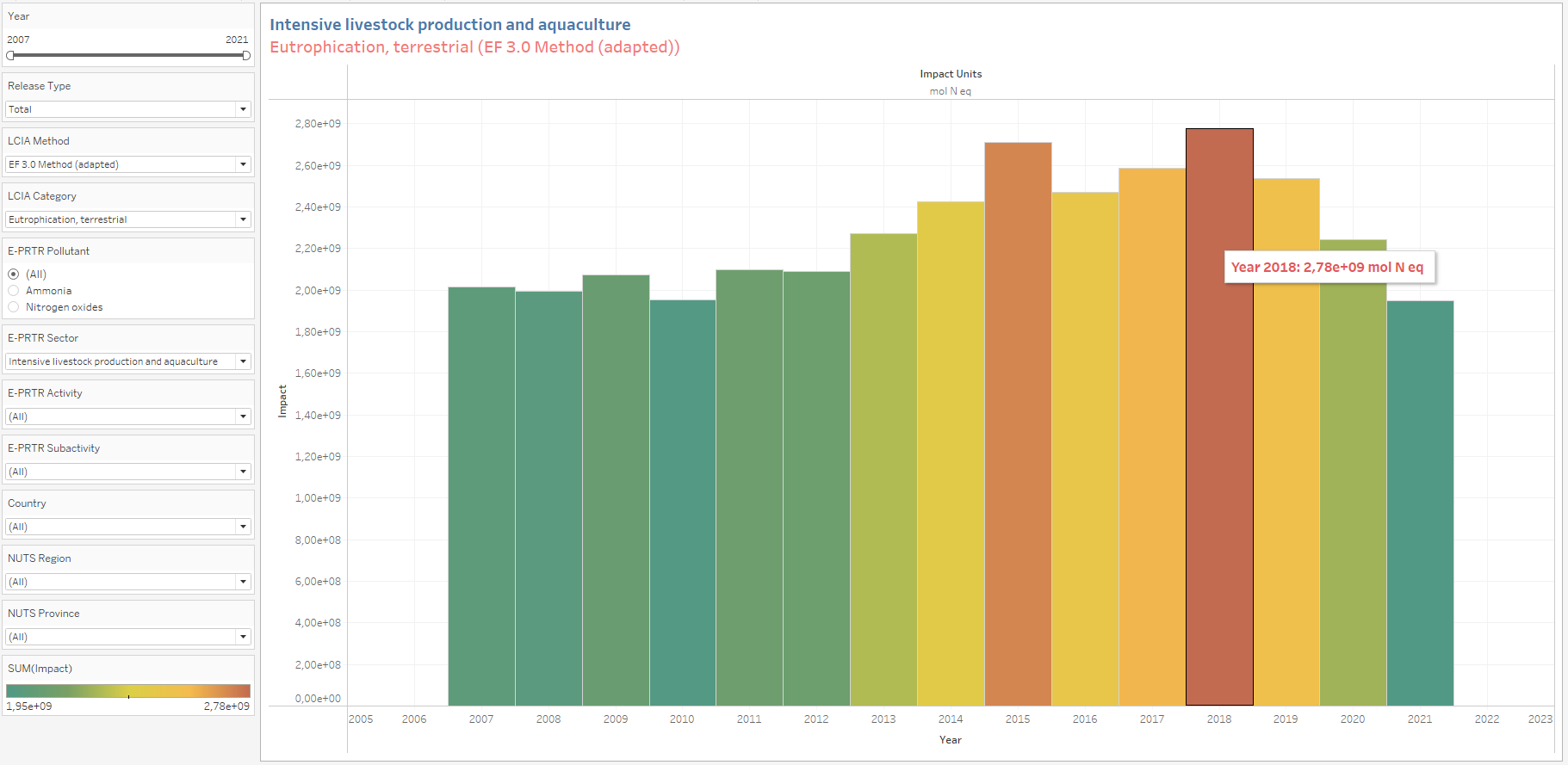

Supplement: Supplementary file 16 — Supplementary file8 (TIFF 249 KB) [file 10661_2024_13565_MOESM8_ESM.tiff]

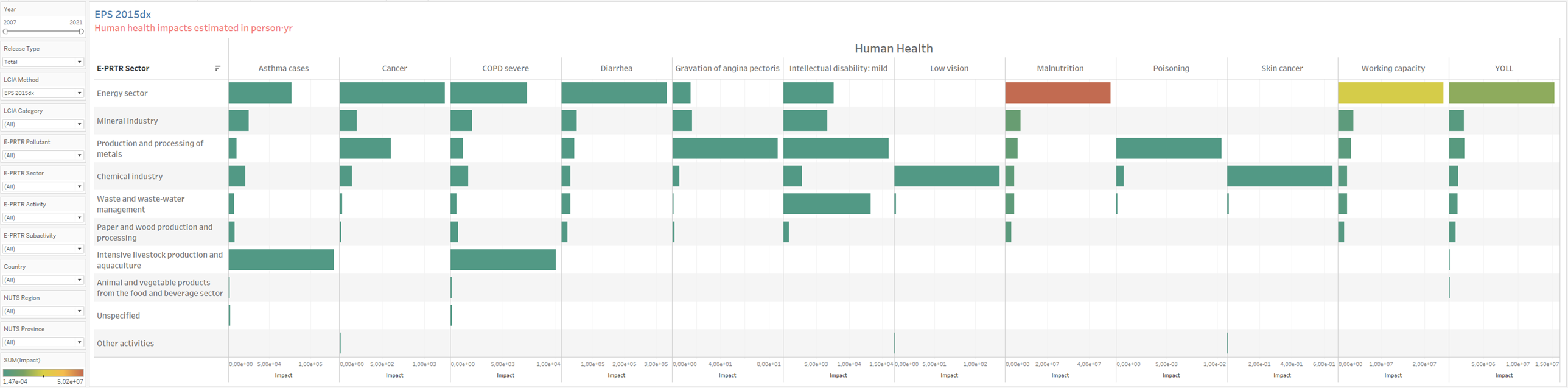

Supplement: Supplementary file 17 — (PNG 1.28 MB) [file 10661_2024_13565_Fig12_ESM.png]

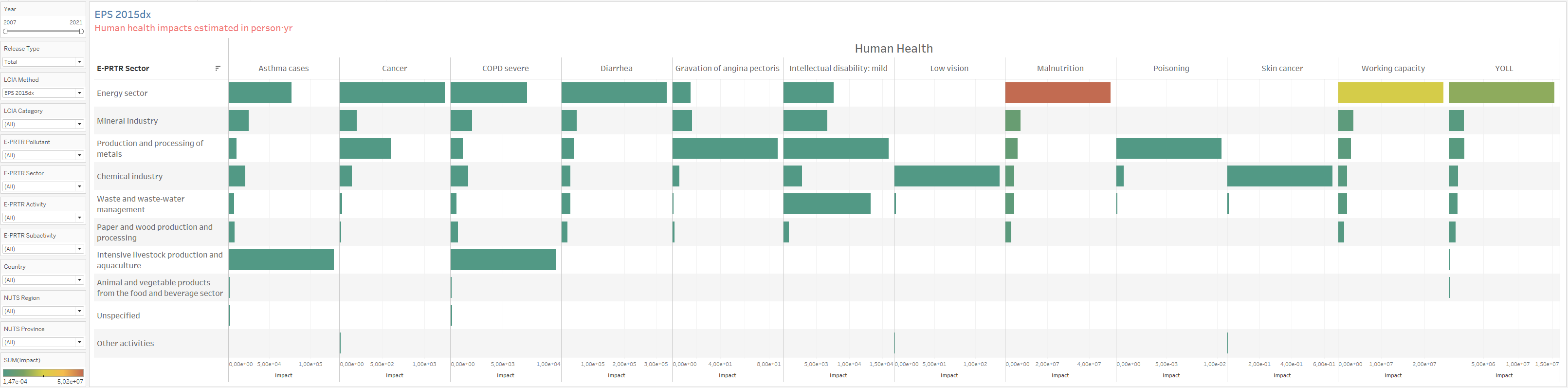

Supplement: Supplementary file 18 — Supplementary file9 (TIFF 338 KB) [file 10661_2024_13565_MOESM9_ESM.tiff]

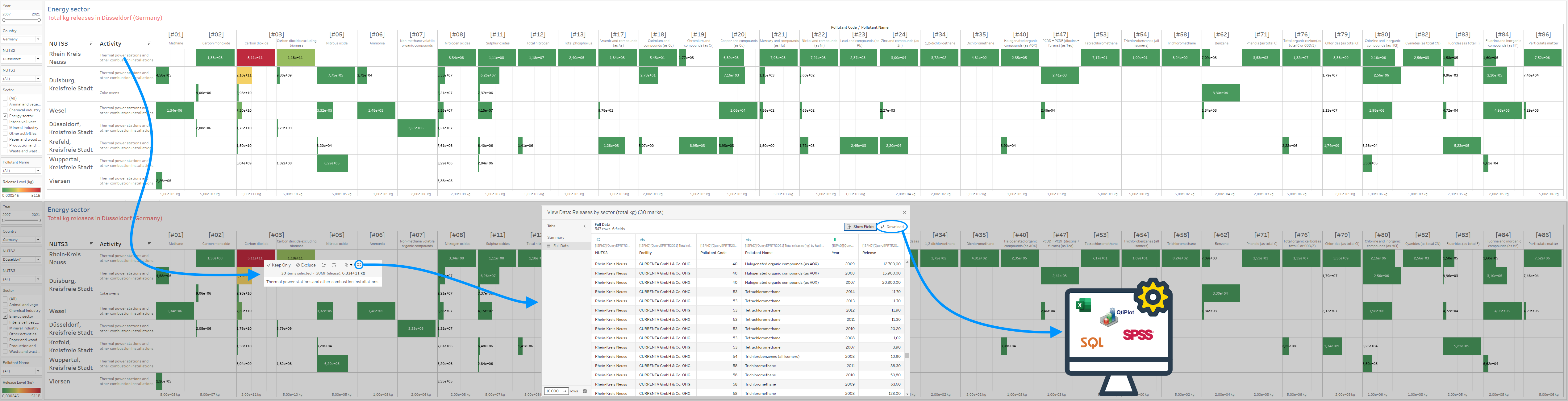

Supplement: Supplementary file 20 — Supplementary file10 (TIFF 1468 KB) [file 10661_2024_13565_MOESM10_ESM.tiff]
